# Combining SNAPs with antibiotics shows enhanced synergistic efficacy against *S. aureus* and *P. aeruginosa* biofilms

**DOI:** 10.1038/s41522-023-00401-8

**Published:** 2023-06-08

**Authors:** Ramón Garcia Maset, Alexia Hapeshi, John Lapage, Niamh Harrington, Jenny Littler, Sébastien Perrier, Freya Harrison

**Affiliations:** 1 Warwick Medical School, University of Warwick, Coventry, CV4 7AL UK; 2 Department of Chemistry, University of Warwick, Coventry, CV4 7AL UK; 3 School of Life Sciences, University of Warwick, Coventry, CV4 7AL UK; 4 Department of Evolution, Ecology and Behaviour, Institute of Infection, Veterinary and Ecological Science, University of Liverpool, Liverpool, L69 7ZV UK; 5 Faculty of Pharmacy and Pharmaceutical Sciences, Monash University, Parkville, Victoria 3052 Australia

**Keywords:** Biofilms, Antimicrobials

## Abstract

Biofilm infections are associated with a high mortality risk for patients. Antibiotics perform poorly against biofilm communities, so high doses and prolonged treatments are often used in clinical settings. We investigated the pairwise interactions of two synthetic nano-engineered antimicrobial polymers (SNAPs). The g-D50 copolymer was synergistic with penicillin and silver sulfadiazine against planktonic *Staphylococcus aureus* USA300 in synthetic wound fluid. Furthermore, the combination of g-D50 and silver sulfadiazine showed a potent synergistic antibiofilm activity against *S. aureus* USA300 using in vitro and ex vivo wound biofilm models. The a-T50 copolymer was synergistic with colistin against planktonic *Pseudomonas aeruginosa* in synthetic cystic fibrosis medium, and this pair showed a potent synergistic antibiofilm activity against *P. aeruginosa* in an ex vivo cystic fibrosis lung model. SNAPs thus have the potential for increased antibiofilm performance in combination with certain antibiotics to shorten prolonged treatments and reduce dosages against biofilm infection.

## Introduction

The ability of bacterial biofilms to bypass the effects of common antibiotics is an increasing concern in the antimicrobial resistance (AMR) crisis^1,2^. Biofilms can survive in various environments (including host-tissues) with high tolerance against antibiotics^3,4^. Biofilms are responsible for more than 80% of chronic infections^5^. In chronic wounds, such as diabetic foot ulcers, biofilms impede wound healing, and increase patient morbidity and mortality^6^. Between 14 and 24% of diabetic foot ulcers will lead to amputation and an elevated mortality risk^7^. In the respiratory system, biofilm infections are the most prominent cause of morbidity and mortality in people with cystic fibrosis (CF)^8^.

Antimicrobial peptides (AMPs) have been recently extensively studied as potential candidates to tackle AMR^9^. This is due to their broad-spectrum activity; their mechanism of action, which is associated with bacterial membrane disruption^10^; and their multiple intracellular bacterial targets, making the emergence of resistance less likely^11^. However, their low stability in physiological conditions, and their cytotoxicity against mammalian cells, hinder their success in clinical trials^12^. Synthetic nano-engineered antimicrobial polymers (SNAPs) have been explored as mimics of AMPs, as they can be designed to overcome some of these drawbacks^13^. The combination of AMPs with standard antibiotics, biocides and other AMPs has also been suggested as a promising strategy to combat bacterial infections. This is because the combination of individual antimicrobials could (1) improve the efficacy of pre-existing treatments, (2) obtain a broad-spectrum antimicrobial activity by the combination of drugs with different mechanisms of action or different targets, and (3) reduce the dosage of the individual drugs to decrease side effects or cytotoxicity in patients^14,15^. Thus, we hypothesised that SNAPs in combination with antibiotics could offer a potent synergistic treatment to combat bacterial biofilms.

## Results

### SNAPs: synergy evaluation against planktonic bacteria

**Fig. 1:**
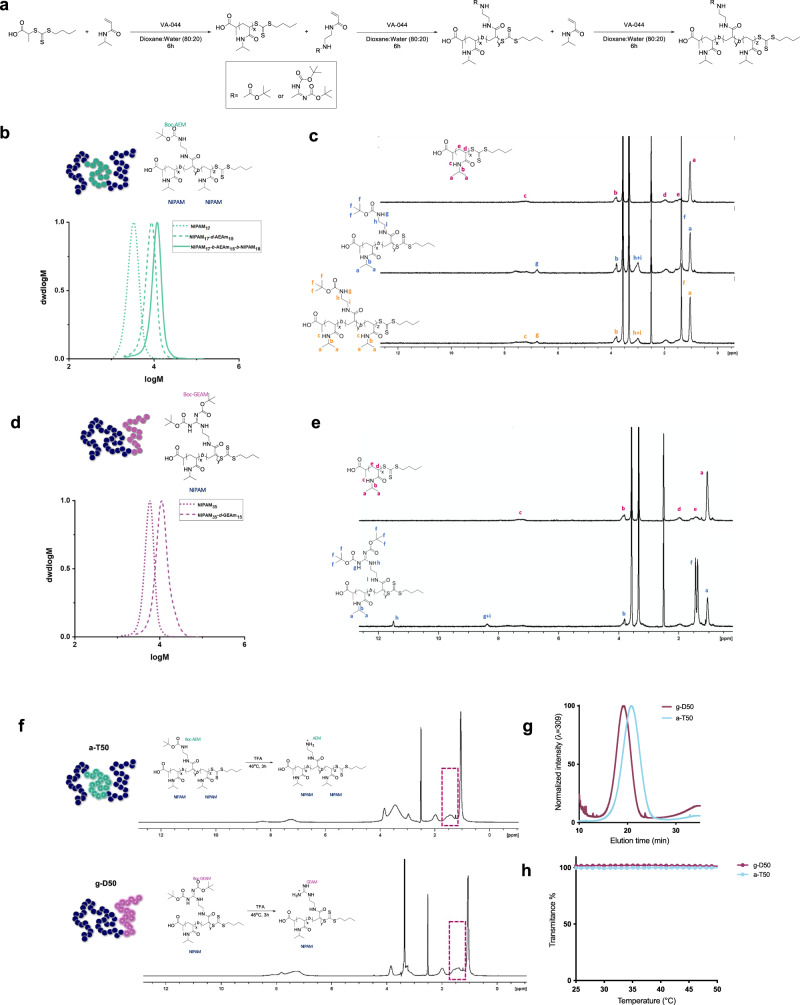
Synthesis and characterization of g-D50 and a-T50 copolymers. **a** General procedure was used for the RAFT polymerisations of the diblock (g-D50) and triblock (a-T50) copolymers. The final degree of polymerization (DP = 50) was kept constant. For ease of reference, the diblock guanidinium copolymer was called g-D50 and the ammonium triblock a-T50. **b** SEC traces in DMF of the Boc-protected a-T50. Dots represent the first block, dashes the first block extension, and solid lines the second block extension. The polydispersity (*Ð*) of a-T50 is 1.15, and the molecular weight (*M*_*n-SEC*_) is 7500 g mol^−1^. **c** ^1^H NMR spectra in DMSO-d6 of the consecutive block extensions to obtain the triblock copolymer a-T50. **d** SEC traces in DMF of the Boc-protected g-D50. The polydispersity (*Ð*) of g-D50 is 1.19, and the molecular weight (*M*_*n-SEC*_) is 10400 g mol^−1^. **e** ^1^H NMR spectra in DMSO-d6 of the consecutive block extensions to obtain g-D50. **f** The Boc removal was confirmed by ^1^H NMR (the red box highlights the disappearance of the signal of the Boc groups). **g** HPLC chromatograms of the copolymers a-T50 and g-D50. **h** UV-vis transmittance at 633 nm for copolymers a-T50 and g-D50.

Two SNAPs were synthesized *via* Reversible Addition-Fragmentation Chain Transfer (RAFT) polymerization for the synergy studies (Fig. 1). The monomers selected in the study are named (guanidino-ethyl)acrylamide (GEAM) and *N*-(2-aminoethyl)acrylamide (AEAM), and their cationic moieties mimic arginine and lysine, respectively. *N*-isopropylacrylamide (NIPAM) was also used to introduce hydrophobicity to the system. A constant ratio of [NIPAM]:[cationic monomer] = 70:30 was maintained to obtain a good antimicrobial activity while reducing the cytotoxic effects as previously reported by Kuroki et al.^16^. The diblock guanidinium copolymer named as g-D50 has been previously reported by our group, showing good antimicrobial activity against *Staphylococcus aureus* and good biocompatibility in vitro and in vivo^17^. In our previous study, we observed that the ammonium triblock SNAP (a-T100-1) showed a potent activity against *Pseudomonas. aeruginosa* affecting both the inner and outer membranes^17^. However, high molecular weight antimicrobial agents might lose the antibiofilm activity due to low penetration into the biofilm matrix^3^. Therefore, we hypothesized that a counterpart of a-T100-1 with lower molecular weight (DP = 50) might improve antibiofilm properties. Consequently, an ammonium copolymer, named as a-T50, having a triblock structure was synthetized (Fig. 1a). As can be observed from the proton nuclear magnetic resonance (^1^H NMR) spectra of both copolymers (Fig. 1c and Fig. 1e), full monomer conversion was reached before chain extension with the second monomer, allowing the synthesis of block copolymers in a one-pot process, without intermediate purification steps. As demonstrated by the size exclusion chromatography (SEC) analysis, the copolymers exhibited monomodal molecular weight distributions with narrow dispersities (Đ ≤ 1.21), indicating good control over the chain extension process (Fig. 1b, d). Subsequently, the SNAPs were deprotected, yielding positively charged copolymers (Fig. 1f). The hydrophobicity of the polymers was evaluated *via* Reverse Phase HPLC (RP-HPLC), following a protocol previously reported by our group^18^, where the guanidinium or ammonium containing copolymers showed a less hydrophobic profile with increasing block segmentation^17^. In this case, a similar trend was observed, with g-D50 eluting first (less hydrophobic) followed by a-T50, in which the hydrophobic block is more segregated, resulting in an increase of the overall hydrophobicity (Fig. 1g). Since PNIPAM is a well-known thermoresponsive polymer, we evaluated the solution behaviour of both copolymers *via* turbidity measurements using UV-vis spectroscopy. The polymer solutions in PBS (pH = 7.4) were prepared at a concentration of 1 mg mL^−1^ and subjected to two heating/cooling cycles from 25 to 60 °C (λ = 633 nm). As can be observed in Fig. 1h, the polymers did not exhibit any thermoresponsive behaviour, as indicated by transmittance values close to 100% over the whole examined temperature range. This suggested that the presence of the positively charged segments increased the overall hydrophilicity of the copolymers, increasing the cloud point temperature of the PNIPAM block to higher values (> 60 °C) or completely hindering its thermoresponsive behaviour. Therefore, the performance of the copolymers in the biological assays will not be influenced by temperature. As previously reported, g-D50 showed an MIC of 64 μg mL^−1^ against *S. aureus* USA300 in cationic-adjusted Mueller-Hinton broth (caMHB) and the compound a-T50 showed potent antimicrobial activity against *P. aeruginosa* PA14 with a value of 32 μg mL^−1^, even more active than the higher molecular weight compound (a-T100-1)^17^.

**Fig. 2:**
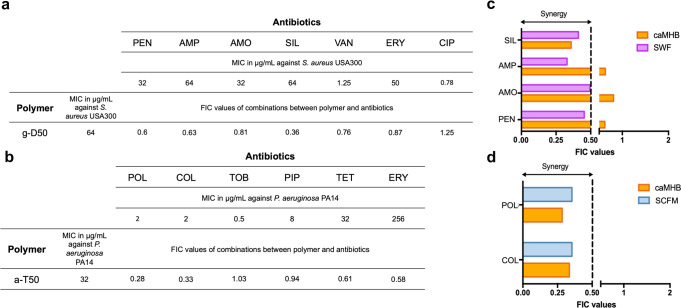
Checkerboard assay: pairwise interaction of copolymers and antibiotics. **a** MIC and FIC values of g-D50 and different antibiotics determined against *S. aureus* USA300 in caMHB. **b** MIC and FICs of a-T50 and different antibiotics determined against *P. aeruginosa* PA14 in caMHB (PEN = penicillin, AMP = ampicillin, AMO = amoxicillin, SIL = silver sulfadiazine, VAN = vancomycin, ERY = erythromycin, CIP = ciprofloxacin, POL = polymyxin B, COL = colistin, TOB = tobramycin, PIP = piperacillin, TET = tetracycline). Three independent experiments (on three different days) were performed. The averages of the three independent experiments were used to calculate the FICs. **c** FIC values of the combination of g-D50 with PEN, AMOX, AMP and SIL against *S. aureus* USA300 in SWF and caMHB media. **d** FIC values of the combination of a-T50 with COL and POL against *P. aeruginosa* PA14 in SFCM and caMHB media. Source data are provided as Supplementary data file 1.

To screen pairwise interactions between drugs, high-throughput checkerboard assays are widely used, with the strength of synergy or antagonism in planktonic culture being formally calculated through the fractional inhibitory concentration (FIC) values^19^. An FIC value < 0.5 indicates synergy: greater antimicrobial activity than the sum of the effects of each individual drug. An FIC value > 0.5 but < 2 indicates an additive or indifferent effect (the antimicrobial effect obtained in the combination is caused by the sum of the two drugs equally, without any potentiation between them). An FIC value ≥ 2 is considered to reflect antagonism, where one drug inhibits or prevents the action of the other^20–22^. We combined g-D50 with several classes of antibiotics or with silver sulfadiazine in checkerboard assays against *S. aureus* USA300 in caMHB^23^. Similarly, we tested the combinatorial activity of a-T50 with several types of antibiotics in a checkerboard assay against *P. aeruginosa* PA14, as summarised in Fig. 2a, b.

The combination of g-D50 with β-lactams (penicillin, ampicillin or amoxicillin), vancomycin, erythromycin or ciprofloxacin resulted in an additive effect against *S. aureus* USA300 with FIC ≥ 0.5 in caMHB. On the contrary, g-D50 and silver sulfadiazine showed a synergistic effect (FIC < 0.5). Media can influence bacterial physiology and antimicrobial susceptibility^24^. We therefore decided to investigate the pairwise combinations in a medium that better reflects the environment encountered in chronic wounds, because these are a common and important infection context for *S. aureus*^25^. Even if β-lactam antibiotics in combination with g-D50 resulted in an additive effect, other studies have reported synergistic effects by combining β-lactams and AMPs against *A. baumanni* in caMHB^26^. We performed the checkerboard assay for the β-lactams and silver sulfadiazine in combination with g-D50 using synthetic wound fluid (SWF) against *S. aureus*^27^. We found synergistic effects for penicillin and amoxicillin in combination with g-D50 in SWF; while g-D50 and silver sulfadiazine showed synergy in both caMHB and SWF (Fig. 2c).

The combinations of a-T50 with polymyxins (polymyxin B and colistin) showed a strong synergistic effect (FIC < 0.5) against *P. aeruginosa* PA14 in caMHB. The rest of the antibiotics tested with a-T50 (piperacillin, tetracycline, erythromycin, and tobramycin) exhibited an additive effect (Fig. 2b). *P. aeruginosa* is the main pathogen associated with cystic fibrosis lung infection^28^, therefore we decided to investigate whether the synergistic effects observed in caMHB were still observed when the bacteria were grown in a medium mimicking the environment in the cystic fibrosis lungs (synthetic CF sputum medium, SCFM)^29^. The results evidenced that the synergistic effect was not altered in the presence of SCFM, and that polymyxins plus a-T50 resulted in synergistic effects in both caMHB and SCFM (Fig. 2d).

**Fig. 3:**
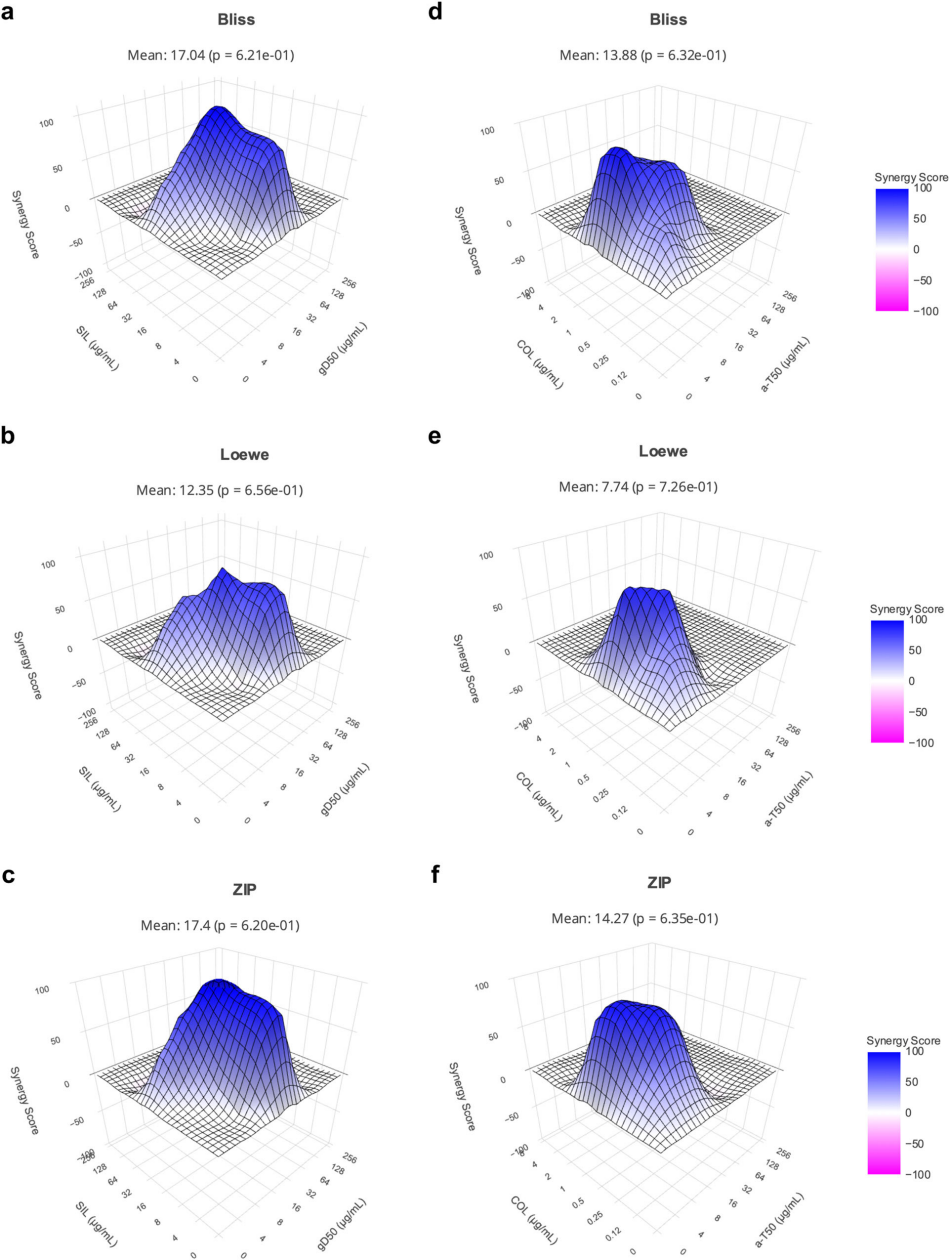
3D synergy landscapes. **a**–**c** 3D synergy plot of g-D50 and silver sulfadiazine against S. *aureus* USA300 in caMHB using the Bliss, Loewe and ZIP models, respectively. **d**–**f** 3D synergy plot of a-T50 and colistin against *P. aeruginosa* PA14 in caMHB using the Bliss, Loewe, and ZIP models, respectively. The synergy profile was calculated using SynergyFinder. The colour gradient from blue to magenta shows the shift of drug interactions from antagonism to synergy score. Source data are provided as Supplementary data file 1.

The most promising synergistic combinations (in both caMHB and the infection-mimicking media) against *S. aureus* USA300 and *P. aeruginosa* PA14 were selected to further investigate the drugs’ interactions. We used SynergyFinder^30^ to calculate the dose-response matrix of the combinations. The dose-response of the drugs and the combinations was investigated by using a resazurin assay (indicating active metabolic bacterial cell activity), since the checkerboard assay cannot distinguish if a drug combination is bactericidal or bacteriostatic. By calculating cell viability using the resazurin assay, the data indicated that the drug interactions caused a bactericidal effect since active metabolic activity was not found (Supplementary Fig. 1), Then, the dose-response of the individual drugs and the combinations were used to create 3D plots of the synergy landscape of the best synergistic combinations (Fig. 3). This offers a better understanding of the drug interactions and dose-relationship between the two drugs than a crude FIC value. The models to predict synergy are based on the drugs’ mechanisms of action (MOA), and can assume the compounds are interchangeable (identical MOA) or have different targets/MOAs^31^. Since multiple MOAs have been reported for AMPs and silver sulfadiazine, it remained unclear whether the drugs can potentially be “interchangeable” or not. In addition, the mechanism of action of colistin has been disputed in the last few years^32^. We therefore explored the synergy landscapes using different statistical models with different assumptions (Loewe, Bliss and Zip model). The Loewe additivity model defines the anticipated effect as if a drug was combined with itself, whereas the Bliss independence model employs probabilistic theory to model the effects of individual drugs in a combination as independent but competing events. The Zero Interaction Potency (ZIP) model is a pharmacological model that combines the Loewe and Bliss models. ZIP assumes that when two non-interacting drugs are administered together, they will have minimal changes in their dose-response curves^33^. The interaction between two drugs is likely to be antagonistic with a synergy score below −10, additive when the synergy score is between −10 and 10, and synergistic when larger than 10^30^. In the case of g-D50 and silver sulfadiazine, the three models indicated a synergy score > 10. In the case of the interaction of colistin and a-T50, Bliss and ZIP models indicated a synergy score > 10. In contrast, in the Loewe model, the synergy score was 7.74, showing an additive interaction.

### Biofilm prevention against *S. aureus* USA300 in the soft tissue wound model

*S. aureus* is commonly found as a biofilm in diabetic ulcers and chronic skin wound infections, causing prolonged antibiotic treatment, recurrent hospitalisation, and limb amputation^34^. To investigate the biofilm prevention potential of g-D50/antibiotic combinations in this context, a soft tissue biofilm wound model, combining SWF with collagen, was used^27^. The SNAPs were inactive against planktonic *P. aeruginosa* using SWF^17^. Hence, we decided to focus on *S. aureus* biofilms in the context of wound infections.

**Fig. 4:**
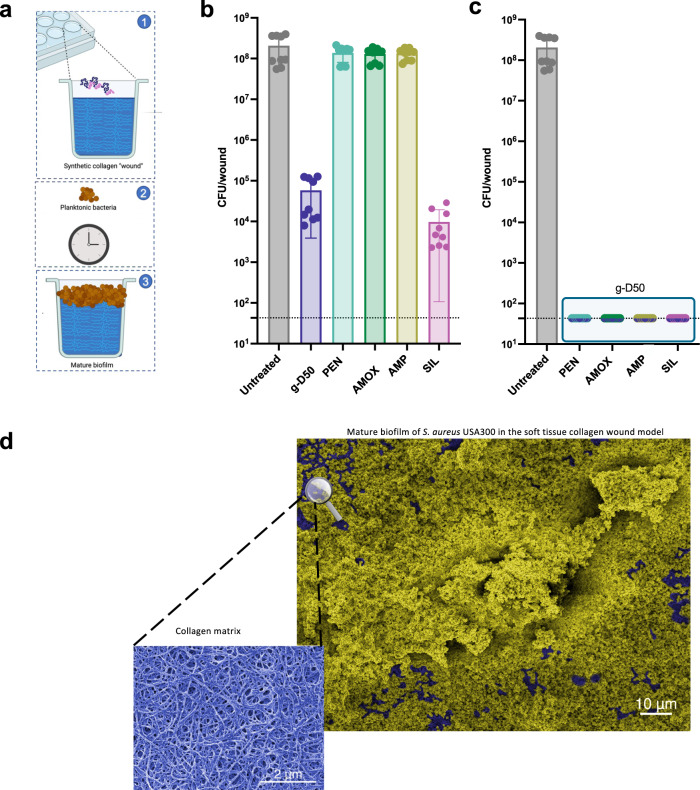
Biofilm prevention assay against *S. aureus* USA300. **a** Schematic describing the biofilm prevention assay in the soft tissue collagen wound model. **b** Effect of single treatments preventing the biofilm formation of *S. aureus* USA300 in the soft tissue wound model. CFU counts (circles) after 48 h of treatment with g-D50, penicillin (PEN), amoxicillin (AMOX), ampicillin (AMP), silver sulfadiazine (SIL) at 2x MIC against *S. aureus* USA300 in the soft tissue wound model. A complete killing was not observed for the individual treatments. A Dunnett’s test was performed to compare the CFU of the individual treatments with the CFU of the untreated controls. g-D50 (2x) and SIL (2x) were significantly different from the respective untreated controls (all *p* < 0.001***, Dunnett’s test). The data were collected from three independent experiments (conducted on different days), using three wounds per treatment. The error bar represents the standard deviation between the replicates. Source data are provided as Supplementary data file 1. **c** CFU counts after 48 h of treatment of g-D50 in combination with PEN, AMOX, AMP and SIL, respectively, at a final concentration of 2x MIC against *S. aureus* USA300 in the soft tissue wound model. The combinations of g-D50 and the respective antimicrobial compounds produced total killing and statistical tests were not performed. The horizontal dashed line represents the limit of detection by plating. **d** SEM images of mature *S. aureus* USA300 biofilm in the soft tissue collagen model. Images were processed with GIMP1-2 for false colouring. The collagen matrix image was obtained from an uninfected wound control, while the image with biofilm was obtained from an infected wound after 24 h. Blue was used to highlight the collagen matrix and yellow was used to highlight *S. aureus* USA300 biofilm.

Artificial wounds were pre-incubated for 10 min with each of the β-lactam antibiotics, silver sulfadiazine or g-D50 individually (2x MIC in SWF) or a combination of each antimicrobial agent with g-D50 (each at 2x MIC in SWF). Untreated wounds (SWF only) were used as controls. Then, *S. aureus* USA300 bacteria were inoculated into the collagen wound and incubated for 48 h at 37 °C as shown in Fig. 4a. β-lactam antibiotics did not show any ability to prevent biofilm formation when used individually. However, both g-D50 (2x MIC) and silver sulfadiazine (2x MIC) had a potent biofilm prevention activity, reducing CFU counts at 48 h by 3-log_10_ and 4-log_10_, respectively, significantly different (*p* < 0.001***, Dunnett’s test) compared to the untreated control (Fig. 4b). Interestingly, the pairwise combination of each of the tested antimicrobials with g-D50 completely inhibited biofilm formation in the collagen model, evidencing synergistic effects on preventing biofilm formation (Fig. 4c). Loewe, Bliss and Zip models could not be calculated since some of the individual treatments did not cause biofilm prevention. The untreated control was visualised using scanning electron microscopy (SEM) to confirm the biofilm formation after 48 h incubation as shown in the false coloured image in Fig. 4d, where yellow indicates the biofilm structure and in blue indicates the collagen matrix.

### Biofilm disruption/eradication against *S. aureus* USA300 in the soft tissue wound model

**Fig. 5:**
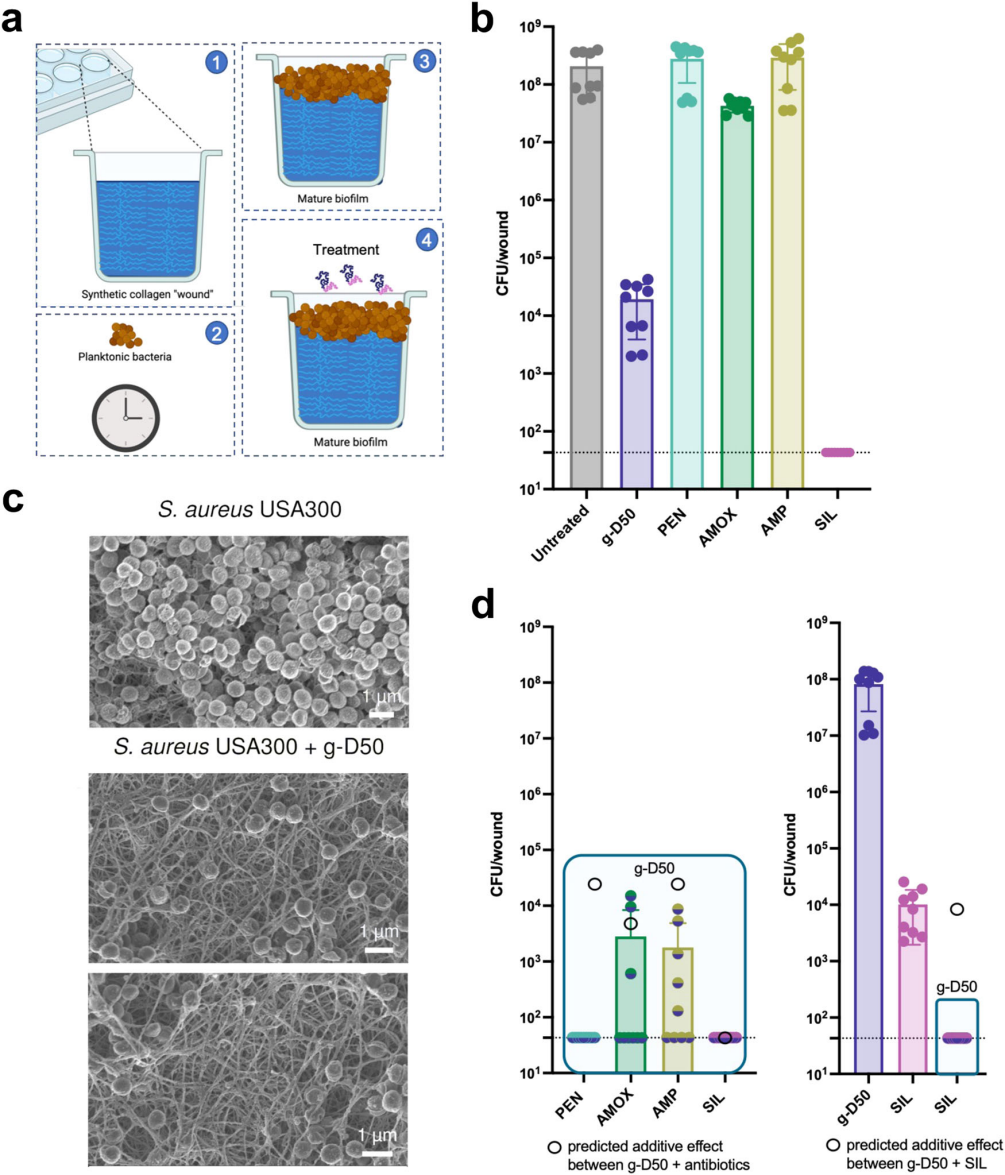
Biofilm disruption assay against *S. aureus* USA300. **a** Schematic representation of the biofilm formation and the following antibiofilm treatment in the collagen wound model. **b** CFU counts (circles) after 24 h of individual treatment of penicillin (PEN), amoxicillin (AMOX), ampicillin (AMP), silver sulfadiazine (SIL) and g-D50 at 10x MIC against mature *S. aureus* USA300 biofilms in the soft tissue wound model. The data were collected from three independent experiments (conducted on different days), each using three wounds per treatment. A Dunnett’s test was performed to compare the CFU of the combination treatments with the CFU of the untreated controls. Only g-D50 (10x MIC) was significantly different from the untreated controls (*p* < 0.001***, Dunnett’s test). SIL (10x MIC) caused complete biofilm eradication and was not included in the statistical analysis. The error bar represents the standard deviation between the replicates. **c** SEM images of (top) mature *S. aureus* USA300 biofilm in the soft tissue collagen model, and (bottom) treated with g-D50 at 10x MIC. **d** CFU counts (circles) after 24 h of combinatorial treatment of g-D50 (10x MIC) with PEN, AMOX, AMP and SIL at 10x MIC in mature *S. aureus* USA300 biofilms in the soft tissue wound model. The data were collected from three independent experiments (conducted on different days), each using three wounds per treatment. T-tests were performed to compare the predicted additive effect (empty black dots) with the CFU counts obtained by the drug combinations. The combination of g-D50 (10x MIC) with AMOX (10x MIC) showed no significant difference in comparison with the additive prediction (t_8_ = −0.634, *p* = 0.5438, t-test). The combination of g-D50 (10x MIC) with AMP (10x MIC) showed lower CFU counts in comparison with the additive prediction (t_8_ = −16.995, *p* < 0.001***, t-test), indicating a synergistic effect. The combination of g-D50 (10x MIC) with PEN (10x MIC) and SIL (10x MIC), respectively, caused a complete biofilm eradication, and no statistical tests were performed. In the case of g-D50 (10x MIC) in combination with SIL (10x MIC), a synergistic effect could not be assumed since the individual SIL (10x MIC) treatment caused complete biofilm eradication. The horizontal dashed line represents the limit of detection by plating. Individual treatments of silver sulfadiazine (SIL) and g-D50 at 5x MIC, and the combinatorial treatment of both compounds at a final concentration of 5x MIC in mature *S. aureus* USA300 biofilms in the soft tissue wound model. The combination of g-D50 with SIL (5x MIC) produced complete biofilm eradication and no statistical test was performed. Source data are provided as Supplementary data file 1.

We next investigated whether the compounds and their combinations could disrupt *S. aureus* USA300 biofilms in the soft tissue wound model (Fig. 5a). The artificial wounds were inoculated with *S. aureus* USA300 and incubated for 24 h at 37 °C to obtain biofilms. Biofilm formation was evaluated using SEM of the surface of the wounds (Supplementary Fig. 2), and SEM plus confocal microscopy of cross-sections of the wounds (Supplementary Fig. 3 and Supplementary Fig. 4). Biofilms were exposed to the antimicrobial agents (single treatments) at 10x MIC, or to SWF as a no-treatment control, as shown in Fig. 5b. β-lactam antibiotics did not show any antibiofilm activity whilst g-D50 exhibited biofilm eradication properties with almost a 4-log_10_ reduction in CFU counts (*p* < 0.001***, Dunnett’s test), which is generally considered clinically relevant^35^. Furthermore, silver sulfadiazine at 10x MIC resulted in almost complete biofilm eradication, as no CFU could be obtained (Fig. 5b). The antibiofilm activity of g-D50 (10x MIC) was further evaluated under SEM. The biofilm structure disappeared after g-D50 treatment, and fewer cells were present in the collagen matrix (Fig. 5c).

To investigate whether combinations of g-D50 with β-lactam antibiotics and silver sulfadiazine had a synergistic antibiofilm effect, we treated 24-h biofilms with pairwise combinations (10x MIC). As the biofilm assays are labour-intensive and require plating out treated biofilms to assess killing by CFU values, they are not readily amenable to standard methods for assessing synergy using checkerboards or time-kill assays. Therefore, some of the antimicrobial agents showed to be inactive against biofilms, and a fractional biofilm inhibitory concentration (FBIC) could not be calculated to evaluate the synergistic effect between the pairwise combinations^36,37^. The Loewe and ZIP models used to analyse interactions in checkerboard assays work with dose responses, and so cannot be used to assess drug interactions in single-dose experiments. However, the Bliss model and the Response Additivity model can be used to assess whether single-dose interactions are additive, synergistic or antagonistic^38^. Under Response Additivity, if drugs A and B act independently (additively), and the fractional change in viable cells caused by A is *p(A)* and the fractional change in viable cells caused by B is *p(B)*, then independent action results in a fractional change in viable cells under combination treatment of *p(A)+p(B)*. i.e. if A causes an *a*log_10_ kill and B causes a *b*log_10_ kill, Response Additivity should produce an (*a* + *b*)log_10_ kill. Synergy would produce a greater than (*a* + *b*)log_10_ kill, and antagonism would produce a less than (*a* + *b*)log_10_ kill. Under the Bliss model, if A and B act independently (additively), and the fractional change in viable cells caused by A is *p(A)* and the fractional change in viable cells caused by B is *p(B)*, then independent action results in a fractional change in viable cells under combination treatment of p(A)+p(B)−[p(A)*p(B)]. This is therefore a slightly less stringent criterion for synergy than the Response Additivity. For this reason, we chose to use the Response Additivity model to test for synergy in our biofilm experiment. A statistical comparison was performed between the predicted CFU in paired treatments under Response Additivity and the actual CFU resulting from paired treatments.

On the one hand, the combination of amoxicillin and g-D50 showed an additive effect on biofilms since no statistical difference was observed with the predictive additive effect (t_8_ = −0.634, *p* = 0.5438, t-test). On the other, the combination of ampicillin and g-D50 showed lower CFU counts in comparison with the additive prediction (t_8_ = −16.995, *p* < 0.001***, t-test), indicating a synergistic effect. The combination of g-D50 and penicillin completely eradicated the *S. aureus* USA300 biofilms, indicating a strong synergistic effect. The combination of silver sulfadiazine and g-D50 also resulted in complete biofilm eradication; however, the same effect was reported for silver sulfadiazine as an individual treatment (Fig. 5d). Hence, we decided to halve the dose of g-D50, silver sulfadiazine and their combination to 5x MIC. As summarised in Fig. 5e, g-D50 at 5x MIC lost its antibiofilm activity while silver sulfadiazine at 5x MIC had a potent antibiofilm activity reducing the CFU counts by 4-log_10_ (*p* < 0.001***, Dunnett’s test). The combination of g-D50 with silver sulfadiazine, each at 5x MIC, completely eradicated the biofilms, suggesting a strong synergistic effect against biofilms. The effect of SIL (5x MIC) and the combinations of g-D50 and SIL (5x MIC) was visualized by SEM (Supplementary Fig. 5), correlating with the obtained CFU counts.

### g-D50 colocalizes within *S.aureus* USA300 biofilms in the soft tissue collagen wound model tissue

**Fig. 6:**
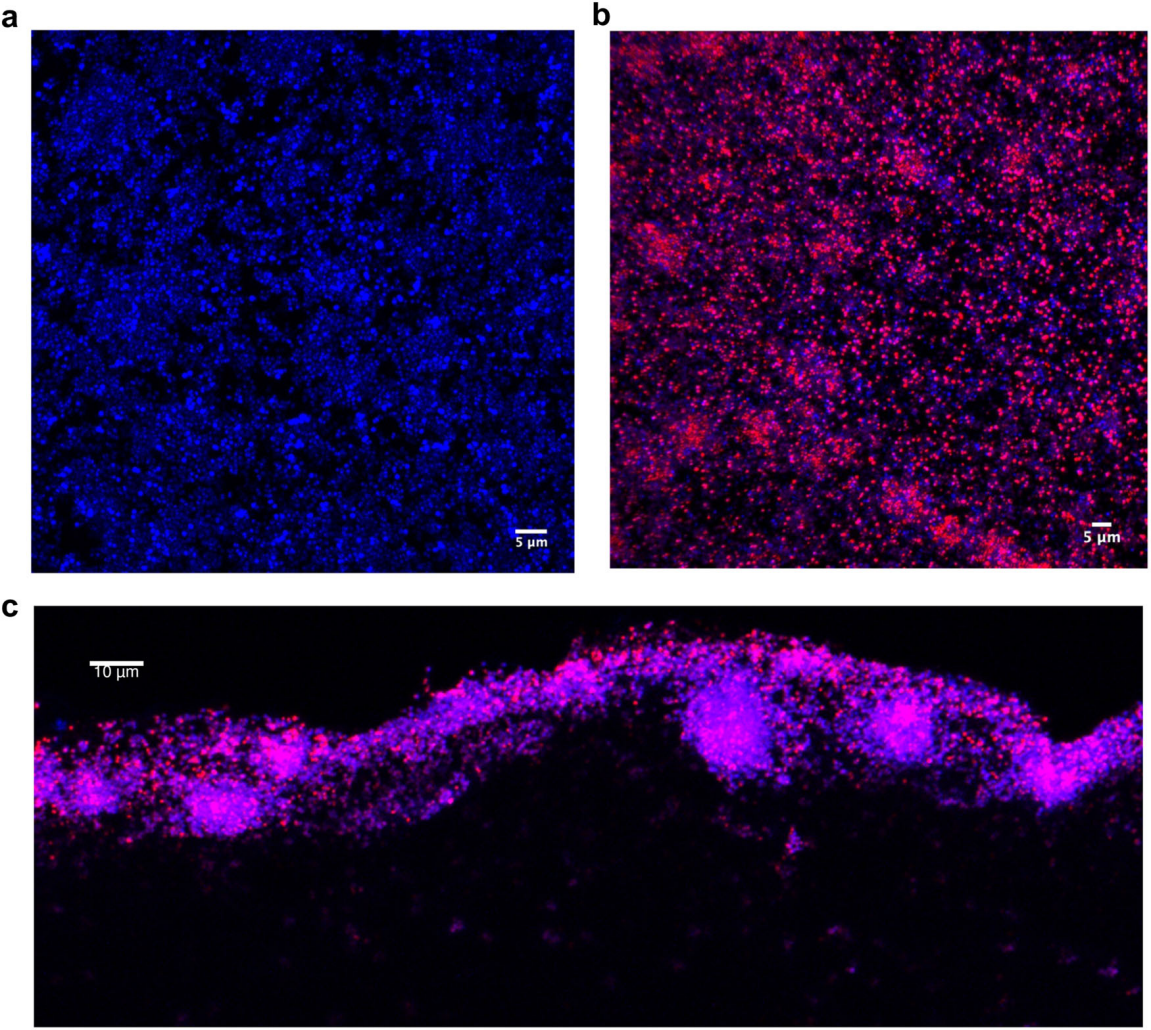
Confocal images of *S. aureus* USA300 biofilm in the soft tissue wound model. **a** Top surface view of *S. aureus* USA300 biofilm in the soft tissue wound collagen model stained with DAPI (blue), visualized by using confocal microscopy after 48 h of infection. **b** Top surface view of *S. aureus* USA300 biofilm in the soft tissue wound collagen model treated with Cy5-g-D50 (magenta) and stained with DAPI, visualized by using confocal microscopy after 48 h of infection. **c** Cross-section view of *S. aureus* USA300 biofilm in the soft tissue wound collagen model treated with Cy5-g-D50 and stained with DAPI, visualized by using confocal microscopy after 48 h of infection.

We sought to investigate whether g-D50 can embed the biofilm matrix as part of its biofilm disruption mechanism. To visualise it, g-D50 was fluorescently labelled with the Cy5 fluorophore as previously reported^17^. A sub-lethal concentration of Cy5-g-D50 (128 μg mL^−1^) was used to assess active diffusion into the *S. aureus* USA300 biofilm in the soft tissue biofilm wound model. After biofilm formation for 24 h, the artificial wounds were treated with Cy5-g-D50 (128 μg mL^−1^ in SWF) or just SWF (negative control) for 24 h, and bacterial DNA was stained with DAPI (1 μg mL^−1^). A reduction of DAPI fluorescent signal in the biofilm samples treated with Cy5-g-D50 was observed in comparison with the untreated biofilm (Fig. 6a, b). This effect might by caused by the cationic charges of g-D50 interacting with bacterial DNA and hindering the DAPI interaction. Another possibility to explain the reduction of DAPI signal could be bacterial cell death, however, the concentration of copolymer used did not cause a reduction in CFU in comparison with the untreated control, so we consider the latter unlikely. To further validate whether the Cy5-g-D50 is able to embed into the biofilm matrix and actively diffuse into the biofilm, a cross-section of the wound model was imaged by confocal microscopy. As can be seen in Fig. 6c and Supplementary Fig. 4, Cy5-gD50 crossed the biofilm matrix and even diffused into the collagen matrix. The colocalization of Cy5-g-D50 into biofilms could explain its antimicrobial activity.

### Synergistic activity against *S. aureus* USA300 biofilm in an ex vivo porcine wound model

**Fig. 7:**
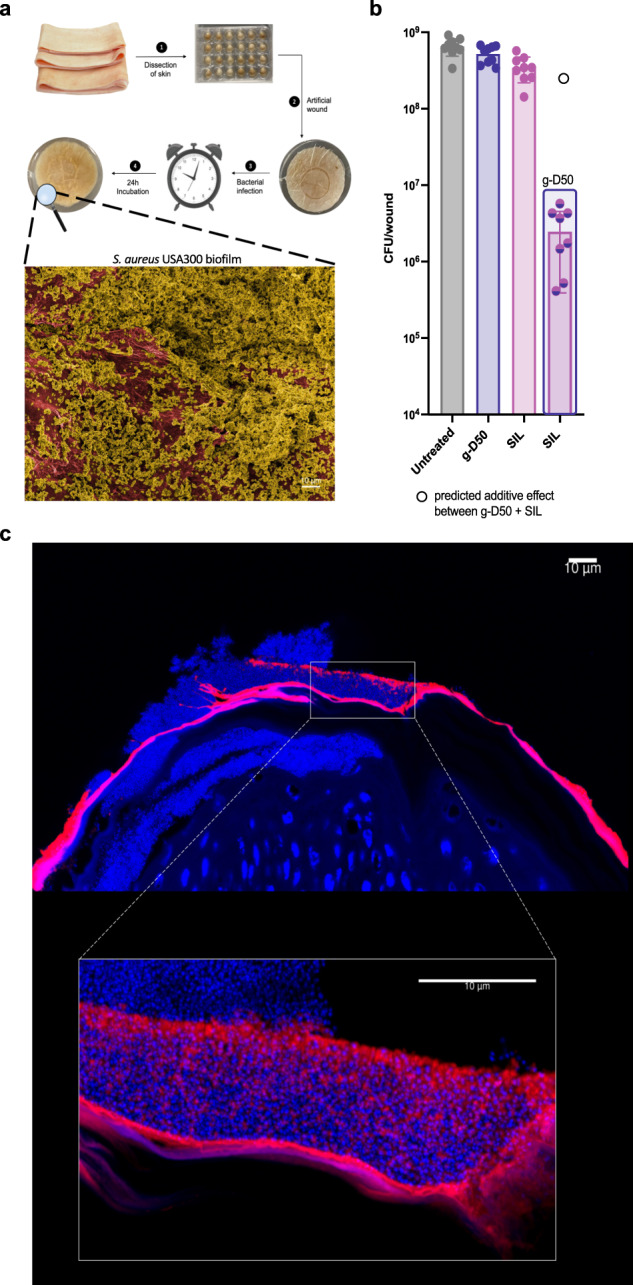
*S. aureus* USA300 biofilm in a porcine skin ex vivo model. **a** Schematic diagram of the porcine skin wound model. Representative electron micrographs of the untreated *S. aureus* USA300 biofilm into the porcine wound model. The image was processed with GIMP1-2 for false colouring. Red was used to highlight the porcine skin and dark yellow was used to highlight the bacteria and biofilm matrix. **b** Biofilm disruption in the porcine ex vivo wound model. *S. aureus* USA300 biofilms were formed for 24 h and subsequently treated with g-D50 and SIL individually at 5x MIC and with the combination of the two drugs at 5x MIC final concentration for 24 h. The data were collected from three independent experiments (conducted on different days), each using three wounds per treatment. A Dunnett’s test was performed to compare the CFU of the individual and the combination treatment with the CFU of the untreated controls, and a significant difference between g-D50 in combination with SIL at 5x MIC and the untreated controls (*p* < 0.001***, Dunnett’s test) was found. The individual treatments of g-D50 (5x MIC) and SIL (5x MIC) were not significantly different from the untreated controls (*p* = 0.3857 and *p* = 0.0698, respectively). The additive effect of the combination of g-D50 (5x MIC) and silver sulfadiazine (5x MIC) was predicted by the sum of the CFU log reductions obtained for each single treatment (indicated with an empty black circle). A t-test was performed to compare the additive effect with the CFU counts obtained by the drug combination. The combination of g-D50 (5x MIC) with SIL (5x MIC) showed lower CFU counts in comparison with the additive prediction (t_8_ = −396.25, *p* < 0.001***, t-test), indicating a synergistic effect against biofilms. The error bar represents the standard deviation between the replicates. Source data are provided as Supplementary data file 1. **c** Representative fluorescent images of the cross-section of the porcine skin wound biofilm (*S. aureus* USA300 biofilm) treated with Cy5-g-D50 (128 μg mL^−1^) in red. DAPI was used to stained nucleic acid (bacterial and mammalian cells) shown in blue. The bottom image is a high magnification of the *S. aureus* USA300 cross-section of the porcine skin model.

The combination of g-D50 and silver sulfadiazine (5x MIC) showed total biofilm eradication in the soft tissue wound model. We sought to investigate whether the pairwise combination maintains biofilm eradication properties in a more realistic ex vivo model of biofilm infection in wounds, that better represents the complex tissue structure found in vivo. The porcine skin was disinfected, dissected and artificial wounds were created as summarized in the diagram of Fig. 7a and described by Yang et al.^39^. The wounds were infected with *S. aureus* USA300 for 24 h with the same bacterial load as the synthetic soft tissue wound model. SEM was used to visualise the formation of biofilm in the porcine model (Fig. 7a and Supplementary Fig. 6). Then, we screened the biofilm eradication activity of g-D50 (5x MIC), silver sulfadiazine (5x MIC), and the combination of both drugs (5x MIC for both agents); SWF was used as an untreated control. The porcine wounds were placed onto SWF agar pads (0.4%) and the corresponding treatment was loaded onto sterile filter paper covering the surface of the wound for 24 h. The individual treatments did not show any antimicrobial effect against biofilms, in agreement with our previous observation in the in vitro synthetic wound model. In the case of silver sulfadiazine, the data showed a discrepancy between the in vitro and ex vivo model, since a potent antibiofilm activity was shown in the in vitro model but not in porcine skin. In general, ex vivo models produce a more robust biofilm that better mimics in vivo characteristics, explaining the higher resistance profiles of biofilm against antibiotics in such models^40^. Nevertheless, the combination of g-D50 and silver sulfadiazine at 5x MIC caused a 2–3-log_10_ killing in the ex vivo skin model, which was a greater effect than the predicted additive effect (Fig. 7b, additive effect indicated by an empty circle). Similarly to the in vitro wound model, we used the Response Additivity model to test for synergy, which indicated lower CFUs in comparison with the predictive effect (t_8_ = −396.25, *p* < 0.001***, t-test).

Following these results, we visualised the active diffusion of the g-D50 into the *S. aureus* USA300 biofilm in the ex vivo wound model. A sub-lethal concentration of Cy5-g-D50 (128 μg mL^−1^) was used to treat the biofilm for 24 h. Then, cross-sections of the porcine wounds were visualised by confocal microscopy. A thick biofilm of approx. 10 μm was observed in the periphery of the skin (Fig. 7c, Supplementary Fig. 7). We observed g-D50 embedded into the biofilm matrix as previously visualized in the collagen model. Interestingly, in the previous model, the g-D50 compound was able to diffuse into the collagen matrix. However, in the ex vivo porcine skin model, Cy5-gD50 was not observed in the skin layer. Limited exposure into the skin might suggest lower cytotoxicity or potential side effects. However, the low penetration could have detrimental effects to treat biofilms in deeper skin tissues.

### Biofilm disruption against *P. aeruginosa* PA14 in an ex vivo pig lung model of cystic fibrosis (EVPL)

**Fig. 8:**
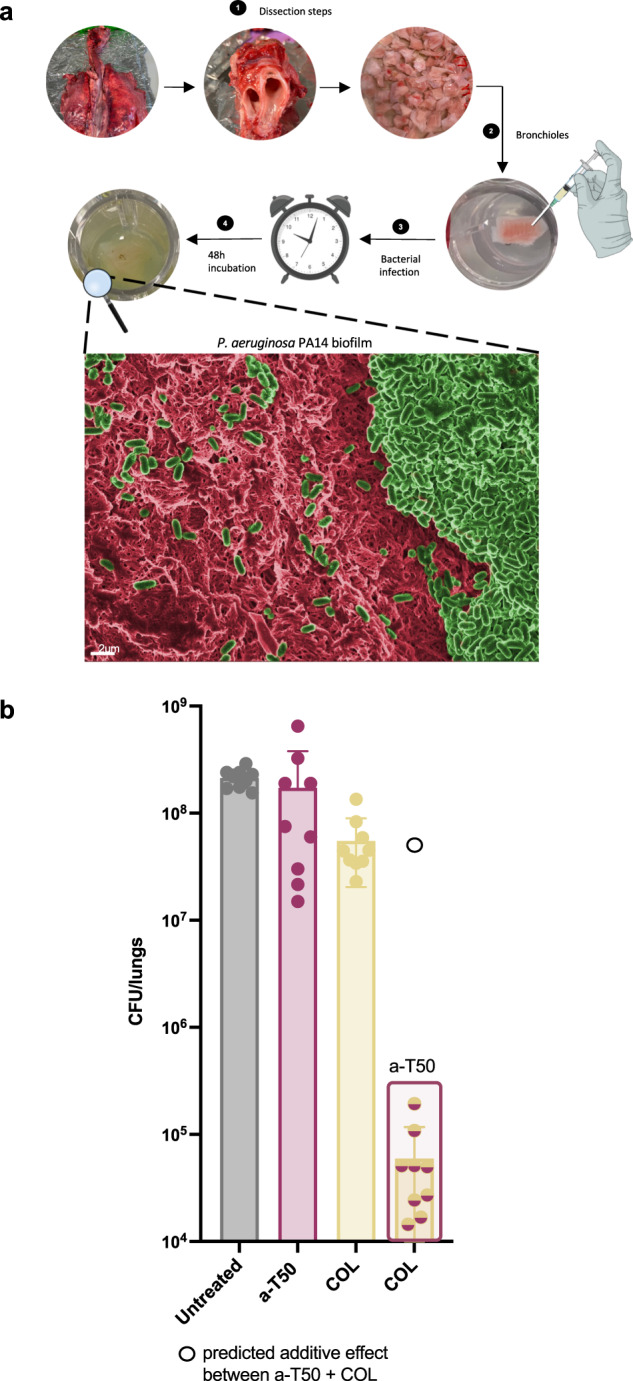
*P. aeruginosa* PA14 biofilm in a pig lung ex vivo model. **a** Schematic diagram of lung dissection for the EVPL model and representative electron micrograph of *P. aeruginosa* PA14 biofilm into pig lung pieces. The image was processed with GIMP1-2 for false colouring. Red was used to highlight the pig lung surface and green was used to highlight the bacteria and biofilm matrix. **b** Biofilm disruption in the EVPL model. *P. aeruginosa* PA14 biofilms were grown for 48 h and subsequently treated with a-T50 and COL individually at 32x MIC and with the combination of the two drugs at 32x MIC final concentration for 24 h. The data were collected from three independent experiments (conducted on 3 different days with 3 different lungs), including three lung pieces per treatment in each experiment. A Dunnett’s test was performed to compare the CFU of the individual treatments with the CFU of the controls. a-T50 (32x MIC) was not significantly different from the untreated control. COL (32x MIC) was significantly different to the untreated control (*p* = 0.00552**, Dunnett’s test). The combination of colistin and a-T50 showed the greatest difference with respect to the untreated control (*p* < 0.001***, Dunnett’s test). The additive effect of the combination of a-T50 (32x MIC) and colistin (32x MIC) was predicted by the sum of the CFU log reduction obtained for each single treatment (indicated with an empty black circle). Then, a t-test was performed to compare the additive effect with the CFU counts obtained by the drug combination. The combination of a-T50 (32x MIC) with COL (32x MIC) showed lower CFU counts in comparison with the additive prediction (t_8_ = −2321.9, *p* < 0.001***, t-test), indicating a synergistic effect against biofilm. The error bar represents the standard deviation between the replicates. Source data are provided as Supplementary data file 1.

The presence of *Pseudomonas* infections in people with cystic fibrosis (CF) is a major cause of morbidity and mortality^41^. We hypothesised that since the SNAP a-T50 showed synergism with colistin against planktonic *P. aeruginosa* in SCFM, the combination could have a synergistic effect against *P. aeruginosa* biofilms. *P. aeruginosa* biofilms were grown in an ex vivo pig lung model (EVPL) that mimics cystic fibrosis infection^42^. Sections of pig bronchiole were dissected and infected with single colonies of *P. aeruginosa* PA14, and used for susceptibility testing following a published methodology^43^. The infected lung pieces were incubated at 37 °C for 48 h to establish biofilms. Lung pieces were visualised by SEM to validate biofilm formation on the lung pieces (Fig. 8a and Supplementary Fig. 8). In a previous study by our group, *P. aeruginosa* clinical isolates showed a high tolerance to colistin in the lung model^44^. Hence, in this study, after biofilm formation for 48 h, the lung pieces were treated with colistin at 32x MIC, a-T50 at 32x MIC, or a mixture of both at 32x MIC. Individually, neither colistin nor a-T50 had antimicrobial activity against *P. aeruginosa* biofilms in the EVPL model. To investigate whether the combinations had a synergistic antibiofilm effect, a predicted additive effect was calculated by summing the CFU reduction caused by colistin and a-T50 individually (indicated by an open circle). Subsequently, a statistical comparison between the CFU predicted under Response Additivity and the obtained CFU was performed. The combination of colistin and a-T50 showed a synergistic effect against *P. aeruginosa* biofilms, as seen in Fig. 8b. Our synergistic combination of colistin with a-T50 caused a clinically relevant 3-log_10_ CFU reduction against *P. aeruginosa* biofilms (t_8_ = −2321.9, *p* < 0.001***, t-test); this effect was not observed for any of the drugs individually. In lung fluids from people with CF, colistin concentration after 12 h drug administration is below 1 mg L^−1^, lower than the clinical MIC against *P. aeruginosa*^45^. In the EVPL model, higher doses of colistin than those reported in CF patient lungs were necessary to obtain 3-log_10_ reduction in CFUs, consistent with the clinical difficulty in eradicating *P. aeruginosa* in CF^44^. Colistin has a limited therapeutic index causing nephrotoxicity^46^, the potential co-administration with a synergistic agent could be very beneficial for patients; further exploration of dose responses to combined agents is needed.

## Discussion

Combining antibiotics with other antimicrobial agents might offer a solution for treating biofilm infections. AMPs have been suggested as potential candidates to target biofilm infections^47^. SNAPs with an improved therapeutic index^48^ might result in superior outcomes, especially if combined with other antimicrobial drugs. The antibiofilm properties of SNAPs have been investigated mainly using simple in vitro biofilm models^49–52^. Few examples of the combination of SNAPs with antibiotics have been reported in the literature^53–56^. As a gold standard methodology, FIC values calculated from checkerboard assays are frequently used as an indication of synergy. However, FIC values vary depending on bacterial species/strains and the media used in the experiments. Using infection-mimicking media could help identify AMPs or synthetic mimics with strong clinical potential: impairment of AMPs’ antimicrobial activity in physiological conditions is one of their main limitations^57^. Our results show that media composition plays an important role in determining the synergistic effect of compounds: for example, penicillin and g-D50 had an additive effect against *S. aureus* USA300 in caMHB and a synergistic effect in SWF.

Furthermore, we used alternatives to FIC, that employ more robust statistical models, to screen the pairwise interactions of our SNAPs with other antimicrobials in checkerboard assays. Synergism landscapes have been exploited to explore anticancer drugs, anti-malarial drugs and antimicrobial agents^58^. Four major synergy models have been described: Loewe additivity (Loewe), Bliss independence (Bliss), highest single agent (HSA) and zero interaction potency (ZIP). To select the adequate synergy model, knowing the MOA of the antimicrobial drug is necessary. To the best of our knowledge, g-D50 and a-T50 target bacterial membranes, causing cell envelope disruption and dissipation of the membrane potential even at sub-MIC concentrations^17^. The action of silver sulfadiazine is caused by both the silver ions and the sulfadiazine. The silver ions interact with the amino acids or carboxylic groups of proteins in the membrane, resulting in damage to the membrane by proton leakage and, subsequently, cell death. Sulfadiazine stops folic acid synthesis (crucial for DNA and RNA) by competitive inhibition of the dihydropteroate synthetase enzyme^59^. Colistin is an antimicrobial peptide, whose mechanism of killing relies on binding to the lipopolysaccharide (LPS) of Gram-negative bacteria^32^. We used the SynergyFinder tool to investigate the synergy landscape between pairs of these agents against clinically-relevant pathogens, using three different statistical models. In all the models, clear synergistic effects were reported, evidencing potentiation between the drugs against planktonic bacteria.

Bacterial biofilms are much harder to kill than planktonic populations. Thappeta et al. reported the synergistic effect of a chitosan-based oligolysine antimicrobial peptide polymer with conventional antibiotics against biofilm wound infection in a mouse model^55^. Therefore, we investigated the antibiofilm properties of selected combinatorial treatments using advanced in vitro and ex vivo biofilm models.

g-D50 disrupted *S. aureus* USA300 biofilms in a synthetic soft tissue wound model. SEM imaging showed a reduction in the biofilm structure and the polymer was able to embed the biofilm matrix in both the in vitro and ex vivo models. The combination of penicillin and g-D50 resulted in total biofilm eradication in the synthetic wound model. Furthermore, the combination of silver sulfadiazine and g-D50 achieved complete eradication of *S. aureus* USA300 biofilm in our collagen in vitro model and a significant CFU reduction in an ex vivo porcine wound model at lower doses of both drugs. The antimicrobial activity of the combination suggests the possibility of treating *S. aureus* biofilm without a large increase in the dosage of each drug, reducing the possible side effects. Silver sulfadiazine formulations have been extensively used in clinics to treat wound infection, as recommended by the UK National Institute for Health and Care Excellence (NICE)^60^. However, cytotoxicity of silver has been reported for burn patients, leading to renal insufficiency and accumulation of silver in the body^61^.

Combinations of certain antibiotics and AMPs have shown synergistic effects against multi-drug resistant *P. aeruginosa* isolates where treatment with single antibiotics was ineffective^62^. In our study, polymyxins and a-T50 had a strong synergistic effect against *P. aeruginosa* in standard caMHB and SCFM. Furthermore, the combination of a-T50 and colistin showed a potent antibiofilm activity against *P. aeruginosa* biofilms in an ex vivo pig lung model of CF lung biofilm. The synergistic combination could reduce the doses of colistin used in clinics, reducing cytotoxicity while maintaining a potent antibiofilm activity.

We note that the labour-intensive nature of the advanced in vitro and ex vivo biofilms models make using “gold standard” synergy assays, such as FBIC, synergy landscape modelling and time-kill assays, costly and time consuming. Our single-dose experiments give a good indication of potential (or not) for synergy, and our results suggest that it would be useful in the future to conduct checkerboard assays or time-kill assays using these models to fully explore the potential for clinically-relevant synergy of penicillin or silver sulfadiazine and g-D50 against *S. aureus* wound biofilms, and of colistin and a-T50 against *P. aeruginosa* in biofilm models.

In conclusion, combinations of SNAPs with antibiotics show a potent synergistic effect to tackle biofilm infections in several advanced biofilm models using clinically important pathogens. Our results underline the fact that pairwise interaction studies need to be carefully designed. This is partly because the effect obtained in vitro against planktonic bacteria might differ depending on the media used, and also because combined effects against biofilm might be completely different from those seen against planktonic cultures. The use of advanced biofilm models to screen for synergy is vital to reduce the risk of failure in subsequent pre-clinical testing and clinical trials.

## Methods

Acetonitrile for HPLC (≥99.9%), acryloyl chloride, bis(*tert*-butoxycarbonyl)-2-methyl-2-thiopseudourea, boc-anhydride (Fluka), chloroform (CHCl_3_), dimethyl sulfoxide-d6 (DMSO, 99.5%), diethyl ether (≥99.9%, inhibitor-free), Dulbecco’s modified Eagle’s medium (DMEM), dichloromethane (DCM), ethanol, ethylenediamine (99%), hexane, magnesium sulfate (MgSO_4_), Müller-Hinton Broth type II (MHB cationic adjusted), *N*-isopropylacrylamide (NIPAM, 97%), *N*,*N*-dimethylformamide anhydrous (DMF 99.8%), paraffin, phosphate-buffered saline (PBS) tablets, polymyxin B sulfate salt, ciprofloxacin, Roswell Park Memorial Institute medium (RPMI-1640), Dublecco’s modified Eagle medium (DMEM), triethylamine (NEt_3_), trifluoro acetic acid (TFA), 1,4-dioxane (≥99) were purchased from Sigma-Aldrich. Ampicillin sodium salt (crystalline powder), amoxicillin (96%), Corning Costar Flat Bottom Cell Culture Plates (Bottom: Flat, Clear, Lid: With Lid, Polystyrene, No. of Wells: 96, Sterile, Surface Treatment: Tissue-Culture Treated), DAPI (4′,6-diamidino-2-phenylindole), Foetal bovine serum (Gibco), Hexamethyldisilazane (Electronic grade, 99+%), Poly-D-Lysine, Silver sulfadiazine (98%), sodium hydroxide pellets, sodium hypochlorite solution (10–15%), SlowFade™ Gold Antifade Mountant, xylene, 2.38 mm metal beads were purchased from Fisher Scientific. Erythromycin, LB agar (acc.to miller for microbiology), Merck Millipore Millicell™ Culture Plate Inserts: PCF, MOWIOL^®^ 4-88 Reagent, penicillin G sodium salt, peptone water, piperacillin, resazurin sodium salt, tetracycline, tobramycin, 24-well plate Corning^®^ Costar^®^ TC-Treated Multiple Well Plates, 48-well plate Corning^®^ Costar^®^ TC-Treated Multiple Well Plates, and sterile homogenisation tubes (BeadBug^™^ unfilled tubes, with caps and sealing ring) were purchased from Merck. Adhesion slides, SuperFrost Plus (631-0448), coverslip (631-0123), round coverslip round of 12 mm (631-1577P), and formaldehyde 4% aqueous solution buffered were purchased from VWR International Ltd (UK). 2′-Azobis[2-(2-imidazolin-2-yl)propane]dihydrochloride (VA-044) was purchased from Wako. Pre-wetted RC tubings 1 kD were purchased from Spectrumlabs. Collagenase type 1 was purchased from EMD Millipore Corp, USA. 29G hypodermic needles were purchased from Becton Dickinson Medical. Breathe-Easier^®^ membrane was purchased from Diversified Biotech. Keyes dermal punch, 2 mm diameter, 10 cm, and Keyes dermal punch, 4 mm diameter, 10 cm were purchased from Surgical Tools. Glutaraldehyde solution 25% for electron microscopy was purchased from PanReac AppliChem. Colistin sulfate salt was purchased from Acros Organics. 2-((Butylthio)-carbonothioyl) thio propanoic acid (PABTC), 1,3-di-Boc-guanidinoethyl acrylamide (diBocGEAM) and *N*-t-butoxycarbonyl-1,2-diaminoethane (BocAEM) were synthesised and purified according to the reported literature^63–65^. The bacterial isolates *S. aureus* USA300 Los Angeles County (LAC) clone and *P. aeruginosa* PA14 were obtained from FH’s laboratory. The porcine skin was purchased from WETLAB LTD, UK and the pig lungs were donated by a local butcher (Quigley and Sons, Cubbington).

### Procedure for the synthesis of the block copolymers via RAFT polymerization

#### Synthesis of the first block

Monomer, initiator (VA-044), CTA (PABTC) and solvents (80% dioxane, 20% water) were introduced in a vial with a magnetic stirrer and a rubber septum. A sample was taken to obtain a t = 0 for ^1^H NMR analysis. The solution was degassed with nitrogen for approximately 20 min. Then, the reaction vial was placed in an oil bath at 46 °C for 6 h to perform the RAFT polymerization. After 6 h, the test tube was withdrawn from the oil bath and a sample was taken for ^1^H NMR and SEC analysis.

#### Synthesis of subsequent blocks

The reaction vial with the reaction mixture was opened and additional monomer, initiator and solvent were introduced. The reaction vial was sealed with a rubber septum and degassed with nitrogen approximately for 20 min. Then, the reaction vial was placed in an oil bath at 46 °C for 6 h to perform the RAFT polymerization. After 6 h, the test tube was withdrawn from the oil bath and a sample was taken for ^1^H NMR and SEC analysis. The quantity of reagents needed for the synthesis of the copolymers is summarized in Supplementary Table 1.

### Deprotection of the Boc groups

TFA was added directly to the polymeric solution in DCM, and stirred for 3 h at 40 °C. After the reaction took place, TFA was removed by precipitation in cold diethyl ether three times. In order to replace the TFA counter-ion, the polymers were dialysed against a NaCl solution, followed by dialysis against distilled water for 3–4 days. Boc-group removal was monitored by ^1^H NMR and ^19^F NMR was used to monitor for traces of the TFA counterion. Finally, the dialysed product was freeze-dried and stored at 4 °C.

### Nuclear magnetic resonance (NMR) spectroscopy

^1^H NMR spectra were recorded on Bruker Avance 300 and 400 spectrometers (300 MHz, 400 MHz, respectively) at 300 K. Data analysis was performed using Mestrenova.

#### Calculation of *M*_*n,th*_

The theoretical number average molar mass (*M*_n,th_) was calculated as

$$M_{{{{\mathrm{n}}}},{{{\mathrm{th}}}}} = \rho M_{{{\mathrm{M}}}}\frac{{\left[ {{{\mathrm{M}}}} \right]_0}}{{\left[ {{{{\mathrm{CTA}}}}} \right]_0}} + M_{{{{\mathrm{CTA}}}}}$$

where [M]_0_ and [CTA]_0_ are the initial concentrations of the monomer and the chain transfer agent, respectively, *ρ* is the monomer conversion as determined by ^1^H NMR, and *M*_M_ and *M*_CTA_ are the molar masses of the monomer and the chain transfer agent, respectively.

### Size exclusion chromatography (SEC)

2 mg mL^−1^ solution of each polymer in DMF containing 0.1% (w:v) LiBr were incubated for 16 h at room temperature. Analyte samples were filtered through a nylon membrane with 0.22 μm pore size before injection. An Agilent PL50 instrument equipped with differential refractive index (DRI) and UV detectors was used for all the measurements. The system was equipped with two x PolarGel M columns (300 × 7.5 mm) and a PolarGel 5 µm guard column, connected in series. The eluent was DMF containing 0.1% LiBr. All experiments were performed at a flow rate of 1 ml min^−1^ at 50 °C. Experimental molar mass (*M*_n,SEC_) and dispersity (*Đ*) values of synthesized polymers were determined by comparison with poly(methyl methacrylate) standards (Agileny EasyVials) using Agilent GPC/SEC software. All data were elaborated using Origin.

### Turbidity measurements

Turbidity analyses for the determination of the transition temperature of each sample were performed using an Agilent Technologies Cary 100 UV-Vis spectrophotometer equipped with an Agilent Technologies Cary temperature controller and an Agilent Technologies 6 × 6 multicell block Peltier. The measurements were performed using Suprasil® quartz cuvettes (Hellman, 100-QS, light path = 10.00 mm) filled with 5 mg mL^−1^ solutions of each polymer in PBS. For each sample, two heating/cooling cycles between 25 and 60 °C were performed with a temperature gradient of 1 °C min^−1^ at λ = 633 nm. All data were recorded using the Cary WinUV software and elaborated using Prism9.

### High-performance liquid chromatography (HPLC)

HPLC was performed using an Agilent 1260 infinity series stack equipped with an Agilent 1260 binary pump and degasser. The flow rate was set to 1.0 mL min^−1^ and samples were injected using Agilent 1260 autosampler with a 20 μL injection volume. The temperature of the column was set at 37 °C. The HPLC was fitted with a phenomenex Lunar C18 column (150 × 4.6 mm) with 5 m packing (100Ǻ). Detection was achieved using an Agilent 1260 variable wavelength detector. UV detection was monitored at λ = 309 nm. Methods were edited and run using Agilent OpenLAB online software and data was analysed using Agilent OpenLAB offline software. Mobile phase solvents used were HPLC grade (ACN was ‘far UV’) and consisted of mobile phase A: 100% ACN, 0.04% TFA; mobile phase B: 100% water, 0.04% TFA with a gradient of 5 to 95% ACN over 30 min. All data was elaborated using Prism9.

### Minimum inhibitory concentrations (MICs)

Minimum inhibitory concentrations (MICs) were determined according to the standard Clinical Laboratory Standards Institute (CLSI) broth microdilution method (M07-A9-2012)^66^. A single colony of bacteria grown on LB agar plates was picked and resuspended in fresh cation-adjusted Mueller-Hinton broth (caMHB), synthetic wound fluid (SWF) and synthetic cystic fibrosis media (SCFM). The concentration of bacterial cells was adjusted by measuring the optical density at 600 nm (OD_600_) to obtain 0.5 McFarland standard (OD_600_ ~ 0.08–0.1) in order to reach a bacterial concentration of ~10^8^ colony-forming units per mL (CFU mL^−1^). The solution was further diluted 100-fold to obtain a concentration of 10^6^ CFU mL^−1^. Polymers were dissolved in their respective medium and 50 μL of each polymer solution was added to wells of a 96-well microplate followed by the addition of the same volume of bacterial suspension, resulting in a final bacterial density of 5 × 10^5^ CFU mL^−1^. The polymer concentration range tested varied from 1024 µg mL^−1^ to 16 µg mL^−1^. The micro-well plates were incubated at 37 °C for 18 h, and growth was evaluated visually. Three independent experiments (three different days) were performed with triplicate wells for each tested concentration for each bacterium and medium. In case of a discrepancy between the biological replicates, the highest MIC value was reported. In addition, MIC experiments were performed using synthetic wound fluid (SWF; peptone water:foetal bovine serum 50:50% v/v), as described by M. Werthén et al. and synthetic cystic fibrosis sputum medium (SCFM), as described by Palmer et al.^27,29^.

### Checkerboard assay: growth determination by resazurin

A single colony of bacteria from LB agar plates was picked and dissolved in fresh caMHB, SFW or SCFM. The concentration of bacterial cells was adjusted by measuring the optical density at 600 nm (OD_600_) to obtain 0.5 McFarland standard (~10^8^ colony forming unit per mL, CFU mL^−1^). The solution was further diluted by 100-fold to obtain a concentration of 1 × 10^6^ CFU mL^−1^. Afterwards, a stock solution of antibiotic and polymeric material was prepared at four times the MIC concentration based on the NCCLS guidelines. In two 96-well plate, 25 μL of caMHB was added into each well. 50 μL of polymeric material was serially diluted along the y-axis in one of the plates leading to a final volume of 25 μL. In the second plate, 50 μL of the second antibiotic was added and diluted along the x-axis leading to a final volume of 25 μL in each individual well. 25 μL of each well of second plate were transferred to first plate, allowing the combination of the polymer and antibiotic at different concentrations with a final volume of 50 μL. This was followed by the addition of the same volume of bacterial suspension, resulting in a final bacterial density of 5 × 10^5^ CFU mL^−1^). The micro-well plates were incubated at 37 °C for 18 h. Subsequently, the bacterial growth was evaluated by adding 10 μL of resazurin dye in each well leading to a final concentration of 0.5 mg mL^−1^. The plates were then incubated for 30 min at 37 °C^67^. A noticeable change of colour could be observed for grown bacteria cells (pink colour) and non-detectable growth (blue colour. In order to determine the type of interaction between polymeric material and antibiotic ΣFICs were calculated. Three independent experiments (on three different days) were performed.

The ΣFICs can be expressed as

$$\Sigma {{{\mathrm{FICs}}}} = \frac{{{{{\mathrm{MIC}}}}_{{{\mathrm{A}}}}\left( {{{{\mathrm{in}}}}\,{{{\mathrm{combination}}}}} \right)}}{{{{{\mathrm{MIC}}}}_{{{\mathrm{A}}}}}} + \frac{{{{{\mathrm{MIC}}}}_{{{\mathrm{B}}}}\left( {{{{\mathrm{in}}}}\,{{{\mathrm{combination}}}}} \right)}}{{{{{\mathrm{MIC}}}}_{{{\mathrm{B}}}}}}$$

The combination is considered synergistic when the ΣFIC is ≤0.5, indifferent when the ΣFIC is >0.5 but <2, and antagonistic when the ΣFIC is ≥2.

### Dose-response matrix and synergy plot

After the incubation of resazurin in the checkerboard assay, the fluorescence signal of the 96-well plate was measured in a Tecan Spark® 10 M plate reader (excitation wavelength = 571 nm; emission wavelength = 584 nm). The bacterial viability in each well was then calculated using the positive control (untreated bacteria) and the negative control or blank (sterile media with resazurin) as follows:

$${{{\mathrm{Bacterial}}}}\,{{{\mathrm{viability}}}} = \left( {\frac{{\lambda _{584}\left( {{{{\mathrm{treatment}}}}} \right) - \lambda _{584}\left( {{{{\mathrm{blank}}}}} \right)}}{{\lambda _{584}\left( {{{{\mathrm{positive}}}}\,{{{\mathrm{control}}}}} \right) - \lambda _{584}\left( {{{{\mathrm{blank}}}}} \right)}}} \right)x\,100$$

The bacterial viability was used as the input in the SynergyFinder^30^. The Loewe, Bliss and Zi models were used to predict the synergy scores. Data were analysed using SynergyFinder^30^.

### Biofilm prevention assay in the synthetic wound model

Synthetic wounds were prepared using a mixture of 2 mg mL^−1^ collagen, 0.01% acetic acid, 60% (v/v) SWF, and 10 mM sodium hydroxide^27^. The mixture was placed on ice and slowly mixed to avoid bubbles. Afterwards, 200 μL of the mixture was transferred to the wells of 48-well microplates. Synthetic wounds were incubated at 37 °C for 1 h, so that the collagen matrix could polymerize. After this incubation period, the 48-well plates were placed under short-wave UV light (Carlton germicidal cabinet) for 10 min to sterilise the wounds prior to infection. Synthetic soft-tissue wounds were then inoculated with 100 μL of polymeric solutions or antibiotic solutions dissolved in SWF, or just SWF as the untreated control. From an agar plate, a few bacterial colonies were diluted into 10 mL of SWF and incubated with shaking at 37 °C for 6 h. Subsequently, the culture was diluted to obtain and OD_600_ of ~0.1–0.2 in SWF. The collagen matrix was infected with 50 μL of the bacterial solution and was incubated at 37 °C for 48 h to allow biofilm formation. Sterile PBS was added to the border wells to avoid any evaporation of the media. After the treatment, 300 μL of collagenase type 1 (0.5 mg mL^−1^) was incubated for 1 h at 37 °C with shaking to break the polymeric matrix of collagen. Solutions from individual wells were obtained and serially diluted to perform bacterial cells counting on LB agar plates. Plates were incubated overnight at 37 °C and colony counts were used to calculate colony forming units (CFU) per wound. Three independent experiments (on three different days) were performed including three artificial wounds per treatment.

### Biofilm eradication assay in synthetic wound model

Synthetic wounds were prepared using a mixture of 2 mg mL^−1^ collagen, 0.01% acetic acid, 60% (v/v) SWF, and 10 mM sodium hydroxide^27^. From an agar plate, a few colonies were diluted into 10 mL of SWF and incubated with shaking for 6 h at 37 °C. The culture was then diluted to obtain and OD_600_ of ~0.1–0.2 in SWF. The collagen matrix was infected with 50 μL of the bacterial solution and was incubated at 37 °C for 24 h to allow biofilm formation. Wounds containing biofilms were then exposed to 100 μL of polymeric solutions and antibiotic solutions dissolved in SWF, or just SWF as the untreated control for 24 h. After the treatment, 300 μL of collagenase type 1 (0.5 mg mL^−1^) was added to the wounds and incubated for 1 h at 37 °C with shaking to break down the polymeric matrix of collagen. Solutions from individual wells were obtained and serially diluted to perform bacterial colony counting on LB agar plates. Plates were incubated overnight at 37 °C and colony counts used to calculate colony forming units (CFU) per wound. Three independent experiments (on three different days) were performed including three artificial wounds per treatment.

### Biofilm eradication assay in an ex vivo porcine skin model

The porcine skin pieces were prepared in sections by shaving and using a biopsy bunch of 14 mm. Artificial wounds were created by using a biopsy bunch of 12 mm and a scalp. Each individual piece of skin was placed in 24-well plates and rinsed with PBS. The surface of the skin was disinfected with a 70% ethanol solution for 30 min, and with a 10% bleach solution for 30 min, followed by a PBS solution with kanamycin (25 μg mL^−1^) and ampicillin (50 μg mL^−1^) incubated overnight. The skin pieces were washed 3 times with PBS. In a 24-well plate, the skin pieces were placed onto 400 μL of SWF solidified with 0.5% (w/v) agarose per well. The plate was then exposed to UV light (Carlton germicidal cabinet) for 5 min. From an agar plate, a few bacterial colonies were diluted into 10 mL of SWF and incubated with shaking at 37 °C for 6 h. Afterwards, the culture was diluted to obtain an OD_600_ of ~0.1–0.2 in SWF. The skin pieces were infected with 10 μL of the bacterial solution and were incubated at 37 °C for 24 h to allow biofilm formation. g-D50 and silver sulfadiazine were dissolved in SWF and 10 μL of the corresponding solution were added to a sterile filter paper (12 mm diameter) and this was placed on top of the skin. Filter paper with sterile SWF was placed on top of the untreated samples to ensure the filter paper did not have an effect on the biofilm. The skin pieces plus filter paper were incubated at 37 °C for 24 h. The ex vivo wounds were then transferred into sterile homogenisation tubes containing eighteen 2.38 mm metal beads and 1 mL of PBS. Samples were bead beaten in a FastPrep-24 5G (MP Biomedicals) for 40 s at 4 m s^−1^ to recover the bacteria from the tissue-associated biofilm. The homogenate solutions were serially diluted in PBS, and 10 µL were spotted onto LB agar plates. Plates were incubated overnight at 37 °C and colony counts were used to calculate colony forming units (CFU) per tissue piece. Three independent experiments (from three different bacterial cultures) were performed with three ex vivo wounds for each treatment per experiment.

### Biofilm killing assay in an ex vivo pig lung model (EVPL) of cystic fibrosis biofilm infection model

Synthetic CF Sputum Medium (SCFM) was prepared as reported by Palmer et al.^29^. The formulation of the media is slightly different in the EVPL model. Glucose was taken out of the original recipe. Fresh porcine lungs were obtained from a local butcher (Quigley and Sons, Cubbington). Briefly, bronchioles were dissected out under sterile conditions and the exterior tissue was removed. The bronchioles were washed once in a 1:1 Dulbecco’s modified Eagle medium (DMEM), RPMI 1640, containing 50 μg mL^−1^ of ampicillin for 15 min. The bronchioles were then sectioned into ~5 mm wide longitudinal strips. The bronchiolar strips were placed in a second 1:1 DMEM:RPMI 1640 and 50 μg mL^−1^ ampicillin wash and cut into squares (~5 × 5 mm). The tissue squares were further washed with 1:1 DMEM:RPMI 1640 and 50 μg mL^−1^ ampicillin. The bronchiolar pieces were then washed with SCFM, UV sterilised for 5 min, and transferred to individual wells of a 24-well plate containing 400 μL of SCFM (solidified with 0.8% (w/v) agarose) per well. The bronchiolar pieces were infected by using sterile 29G hypodermic needles. From an LB agar plate of *P. aeruginosa* PA14, a single colony was touched with the needle and the bronchiolar pieces were pierced. Afterwards, 500 μL of SCFM was added to each well. The 24-well plate was sealed with a Breathe-Easier® membrane and incubated at 37 °C for 48 h. Infected lung pieces were removed from the 24-well plate following incubation, and each piece was briefly washed in 500 μL of PBS. Tissue pieces were then transferred into sterile homogenisation tubes containing eighteen 2.38 mm metal beads and 1 mL of PBS. The issues were bead beaten in a FastPrep-24 5 G (MP Biomedicals) for 40 s at 4 m s^−1^ to recover the bacteria from the tissue-associated biofilm^68^. Three independent experiments (from three different lungs in different days) were performed with three ex vivo bronchiole pieces for each treatment per experiment. To determine the bacterial load, the homogenate solutions were serially diluted in PBS and plated on LB agar. Plates were incubated overnight at 37 °C, and colony counts were used to calculate colony-forming units (CFU) per tissue piece.

### Scanning electron microscopy imaging of biofilms

#### Surface and cross-section imaging of biofilms in the synthetic wound collagen model

Synthetic wounds were prepared using a mixture of 2 mg mL^−1^ collagen, 0.01% acetic acid, 60% (v/v) SWF, and 10 mM sodium hydroxide^27^. In 24-well plates, Millicell® culture plate inserts were used as supports to form the artificial collagen wounds. The insert could be easily removed to obtain the collagen wound for SEM imaging. Samples were incubated with a 2.5% glutaraldehyde solution in PBS (0.5 mL) for 1 h, which was then discarded, and the samples were rinsed 3 times with PBS. For the cross-section imaging, after fixation and washing, the inserts were removed from the 24-well plate and the membrane of the insert was pierced with a scalpel and the collagen wound was removed. With the help of two scalpels, a cross-section was carefully obtained and placed in a cover slide. For the samples into the inserts and the cross-section samples, incubation with a gradient of ethanol baths (from 20%, 50%, 70%, 90%, 100%, and 100%) was performed for 10 min at each ethanol concentration. In both cases, after complete dehydration, the samples were moved to clean wells and were incubated with 0.5 mL of hexamethyldisilazane (HDMS) for 30 min. The HDMS solution was then discarded, and the inserts/cover slides were left to dry in a laminar flow cabinet for 1 h. With the help of a scalpel, the membrane of the insert was carefully removed to obtain the artificial collagen wound. Subsequently, copper tape was added to SEM sample holders and the samples were placed on top. Finally, the samples were sputtered with carbon and immediately analysed using a Zeiss Gemini Scanning Electron Microscope equipped with an InLens detector, at a voltage of 1 kV. All data were analysed using Omero5.6^69^.

#### Surface imaging of biofilms in the porcine skin biofilm model

The porcine wound model samples were fixed with 0.5 mL of a 2.5% glutaraldehyde solution (PBS) overnight at 4 °C in a 24-well plate. After fixation, the 2.5% glutaraldehyde solution was discarded, and the samples were rinsed 3 times with PBS. The porcine wound model samples were moved to clean wells and dehydration was performed using an ethanol gradient (from 20%, 50%, 70%, 90%, 100%, and 100%) for 1 h at each concentration. After complete dehydration, the samples were moved to clean wells and were incubated with 0.5 mL of hexamethyldisilazane (HDMS), as the drying agent, for 3 h. The HDMS solution was then discarded, and the samples were moved to clean wells and left to dry in a flow laminar cabinet for 1 h. The following steps and imaging were performed as previously described in the SEM section. All data were elaborated using analysed.6^69^.

### Confocal microscopy in the biofilm models

#### Surface imaging of biofilm in the synthetic wound collagen model

After 24 h of biofilm formation, the wounds were treated with 25 μL of Cy5-g-D50 (16 μg mL^−1^) for 24 h. Subsequently, the biofilms were rinsed carefully with PBS twice. The biofilm-wound samples were stained with DAPI for 10 min in the dark (5 μg mL^−1^), followed by 3 washes with PBS. The biofilms were then fixed using a 4% formaldehyde solution in PBS for 30 min and rinsed 3 times with PBS. Synthetic wounds were prepared for microscopy as “coffin slides”^70^. Briefly, 7 layers of masking tape were applied to a SuperFrost Plus Slide (631-0108) and a well cut out with a razor of the same thickness of the sample was prepared and the sample was inserted in the “coffin”. 50 µL of Mowiol was pipetted into the well and the synthetic wound floated within. Additional Mowiol was added until the well was full, and a 22 × 22 mm coverslip (631-0123) was applied. The slides were left to set overnight in the dark, and then sealed with nail varnish. All data were analysed using Omero5.6^69^.

#### Cross-section imaging of biofilms in the synthetic wound model

After 24 h of biofilm formation, the wounds were treated with 25 μL of Cy5-g-D50 (16 μg mL^−1^) for 24 h. The biofilms were then rinsed carefully with PBS twice. The biofilm-wound samples were stained with DAPI for 10 min in the dark (5 μg mL^−1^), followed by 3 washes with PBS. Subsequently, the biofilms were fixed using a 4% formaldehyde solution in PBS for 2 h and rinsed 3 times with PBS. The samples were then embedded in paraffin following the protocol described by Johannson et al.^71^. In short, samples were immersed in a series of organic solvents for brief periods of time, followed by three incubations in melted paraffin wax. The series consisted of two 30-min incubations in methanol, two 20-min incubations in 100% ethanol, two 15-min incubations in xylene, and two 30-min incubations in warm paraffin, followed by a final 45-min incubation in warm paraffin. The temperature of the paraffin was maintained at a level just above its melting point. After the paraffin infiltration, samples were embedded in blocks and sectioned using a Hyrax M25 Microtome. Ribbons of 10 μm were produced and mounted on Superfrost Plus slides (631-0108). Sections were then stored at room temperature until imaging. Imaging was performed on a Zeiss LSM 880 fluorescence microscope. The 100× oil objective was used together the AiryScan detector. All data were analysed using Omero5.6^69^.

#### Cross-section imaging of biofilm in the porcine skin biofilm model

The porcine wound model samples were fixed with 0.5 mL of a 2.5% glutaraldehyde solution (PBS) overnight at 4 °C in a 24-well plate. After fixation, the 2.5% glutaraldehyde solution was discarded, and the samples were rinsed 3 times with PBS. After the fixation, the embedding and the cross-section were prepared as described in “Cross-section imaging of biofilms in the synthetic wound model”. Imaging was performed on a Zeiss LSM 880 fluorescence microscope. The 100× oil objective was used together the AiryScan detector. All data were analysed using Omero5.6^69^.

### Statistical analysis

The statistical analyses were performed with R version 3.6.3 (R Development Core Team, 2020) using the multcomp, stats and readxl packages^72–74^. In the case of “biofilm prevention against *S. aureus* USA300 in the soft tissue wound model” for individual treatments, the data were square-root transformed to meet assumptions of linear modelling. The ANOVA test showed significant differences between treatments (F_5,36_ = 1148.8, *p* < 0.001***), between the independent experiments (F_2,36_ = 55.91, *p* = < 0.001***) and the effect of treatment was not significantly different in the independent experiments (F_8,36_ = 0.930, *p* = 0.507).

In the case of “biofilm eradication against *S. aureus* USA300 in the soft tissue wound model” for individual treatments, the data were log-transformed to meet the assumptions of linear modelling. The ANOVA test showed significant differences between treatments (F_4,40_ = 175.9, *p* < 0.001***).

In the case of “biofilm eradication using the porcine skin model”, we used a generalised linear model with a gamma distribution because the data could not be transformed to meet the assumptions of ANOVA. There was a significant difference between the treatments and untreated control ($X_{35}^2$ = 19.112, *p* < 0.001***).

In the case of “biofilm killing assay in an ex vivo pig lung model (EVPL) of cystic fibrosis biofilm infection model”, The data were log-transformed to meet assumptions of linear modelling. The ANOVA test showed significant differences between treatments (F_3,24_ = 465.16, *p* < 0.001***).

## Supplementary information

Supplementary Information

Supplementary data set 1

## Data Availability

The data that support the findings (including the statistical analysis) of this study are available in the main article, Supplementary information files or from the corresponding author upon reasonable request. The source data underlying Figs. 2, 3, 4b, 5b, d, 7b, 8b Supplementary Fig. 1 are provided as Supplementary data file 1.
