# Author Correction: Combining SNAPs with antibiotics shows enhanced synergistic efficacy against *S. aureus* and *P. aeruginosa* biofilms

**DOI:** 10.1038/s41522-023-00407-2

**Published:** 2023-06-16

**Authors:** Ramón Garcia Maset, Alexia Hapeshi, John Lapage, Niamh Harrington, Jenny Littler, Sébastien Perrier, Freya Harrison

**Affiliations:** 1 Warwick Medical School, University of Warwick, Coventry, CV4 7AL UK; 2 Department of Chemistry, University of Warwick, Coventry, CV4 7AL UK; 3 School of Life Sciences, University of Warwick, Coventry, CV4 7AL UK; 4 Department of Evolution, Ecology and Behaviour, Institute of Infection, Veterinary and Ecological Science, University of Liverpool, Liverpool, L69 7ZV UK; 5 Faculty of Pharmacy and Pharmaceutical Sciences, Monash University, Parkville, VIC 3052 Australia

**Keywords:** Biofilms, Antimicrobials

Correction to: *npj Biofilms and Microbiomes* 10.1038/s41522-023-00401-8, published online 08 June 2023

In this article the funding from MRC Doctoral Training Partnership [grant number MR/N014294/1] was omitted. The original article has been corrected.

