# Supplementary material for: Combining SNAPs with antibiotics shows enhanced synergistic efficacy against S. aureus and P. aeruginosa biofilms
Source: NPJ Biofilms Microbiomes. 2023 Jun 8;9:36. doi: 10.1038/s41522-023-00401-8 (PMC10250483; doi:10.1038/s41522-023-00401-8)
Supplement: Supplementary file 1 — Supplementary Information [file 41522_2023_401_MOESM1_ESM.pdf]

Supplementary information

Combining SNAPs with antibiotics shows enhanced synergistic efficacy against *S. aureus* and *P. aeruginosa* biofilms

Ramón Garcia Maset*[1], Alexia Hapeshi[2], John Lapage[3], Niamh Harrington[4], Jenny Littler[3], Sébastien Perrier*[1,2,5] , Freya Harrison*[3]

[1] Warwick Medical School, University of Warwick, Coventry, CV4 7AL, UK
[2] Department of Chemistry, University of Warwick, Coventry, CV4 7AL, UK
[3] School of Life Sciences, University of Warwick, Coventry, CV4 7AL, UK
[4] Department of Evolution, Ecology and Behaviour, Institute of Infection, Veterinary and Ecological Science, University of Liverpool, L69 7ZV, UK
[5] Faculty of Pharmacy and Pharmaceutical Sciences, Monash University, Parkville, Victoria 3052, Australia

r.maset@ucl.ac.uk

S.Perrier@warwick.ac.uk

F.Harrison@warwick.ac.uk

Supplementary Table 1. Reaction conditions of the RAFT polymerizations.

	Polymer composition	$m_{monomer1}$ (mg)	$m_{monomer2}$ (mg)	$m_{monomer3}$ (mg)	M_{CTA} (mg)	$[CTA]_0/]VA\text{-}044]$ 1st	$[CTA]_0/]VA\text{-}044]$ 2nd	$[CTA]_0/]VA\text{-}044]$ 3rd	V_{final} (µL)
g-D50	$NIPAM_{35}$-*b*-$GEAM_{15}$	227.86	306.21	-	13.75	10	14	-	2140
a-T50	$NIPAM_{17}$-*b*-$AEAM_{15}$-*b*-$NIPAM_{18}$	114.41	190.32	119.76	14.02	10	14	10	2670

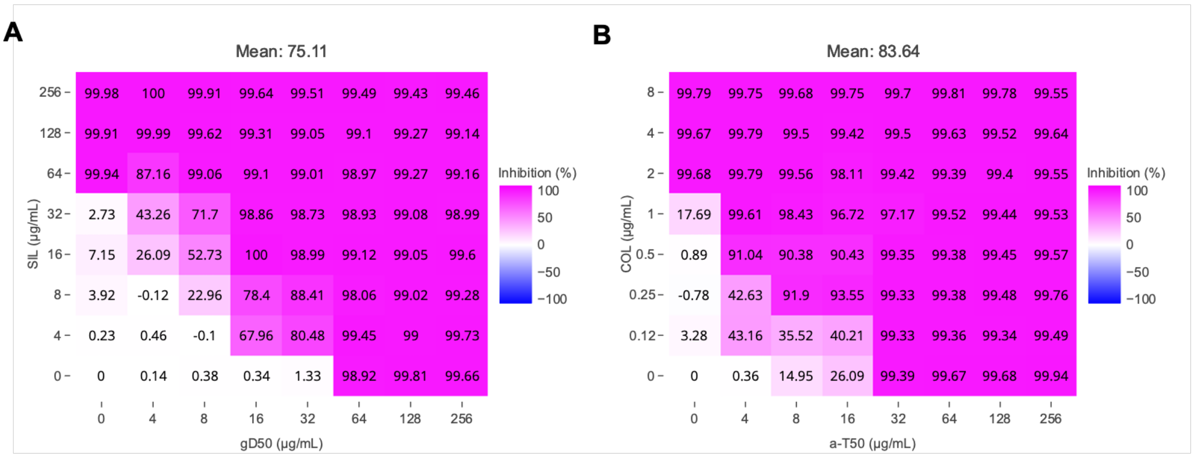

Supplementary Figure 1. (A) Dose-response matrix of the pairwise combination of g-D50 and silver sulfadiazine against *S. aureus* USA300 in caMHB and **(B)** dose-response matrix of the pairwise combination of a-T50 and colistin against *P. aeruginosa* PA14 in caMHB. The increase in the magenta colour gradient denotes an increase in the percentage of inhibition of the different pairwise combinations. Data were plotted and analysed by using SynergyFinder.

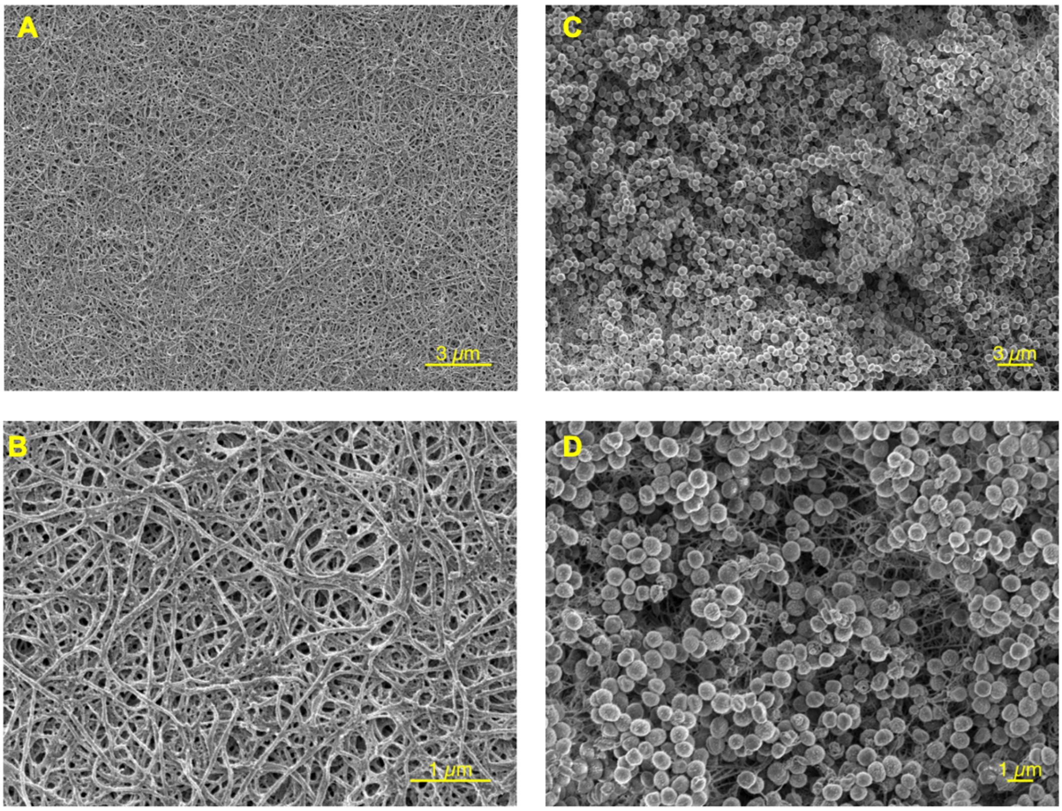

Supplementary Figure 2. Representative SEM micrographs of **(A-B)** uninfected collagen matrix, and **(C-D)** biofilm of *S. aureus* USA300 in the collagen matrix after 48 h incubation.

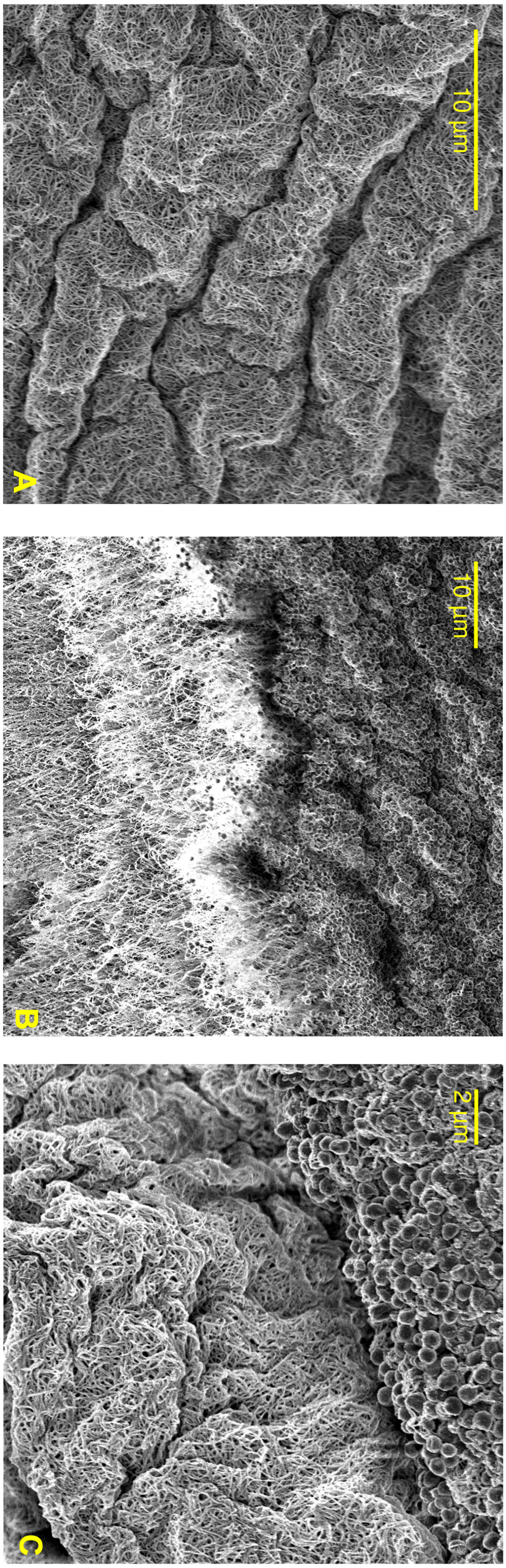

Supplementary Figure 3. SEM micrographs of **(A)** the cross section of uninfected collagen matrix, and **(B-C)** the cross section of biofilm of *S. aureus* USA300 in the collagen matrix after 48 h incubation.

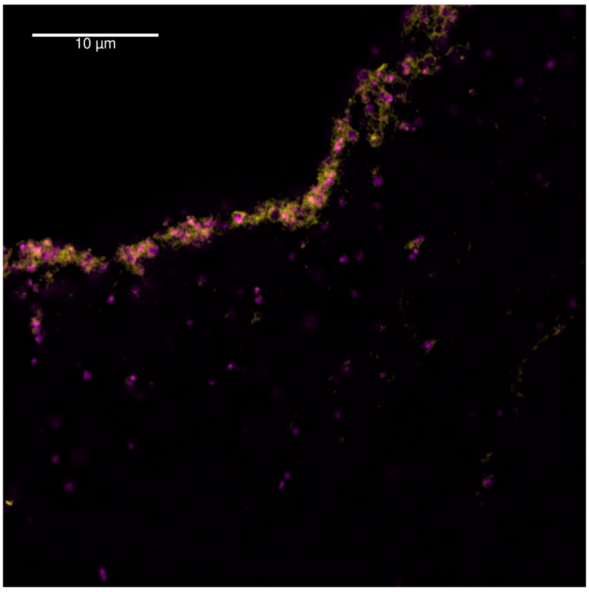

Supplementary Figure 4. Cross section view of the *S. aureus* biofilm in the soft tissue wound collagen model treated with Cy5-gD50 (magenta) and stained with calcofluor (yellow) that stains the biofilm matrix, visualized by using confocal microscopy after 48 h of infection

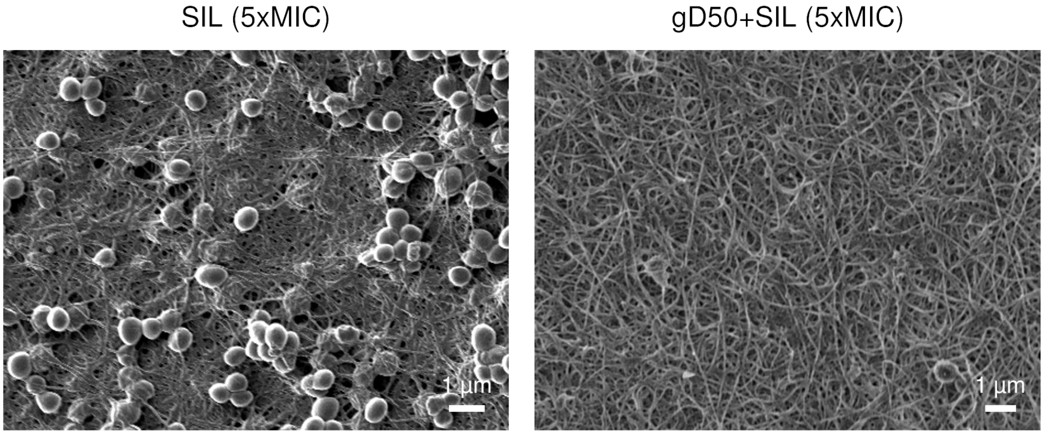

Supplementary Figure 5. SEM micrographs of *S. aureus* USA300 into the biofilm model treated with SIL (5xMIC) and the combination of SIL + gD50 (5xMIC).

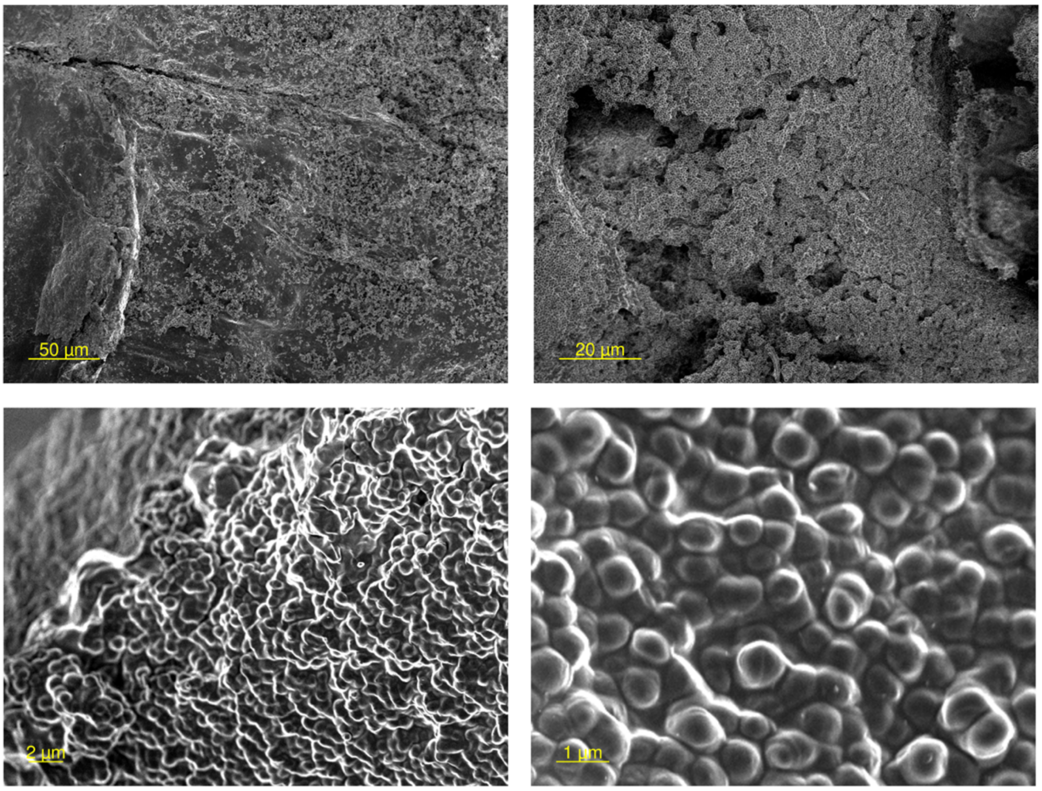

Supplementary Figure 6. Representative SEM micrographs of *S. aureus* USA300 biofilm in the *ex vivo* porcine wound model.

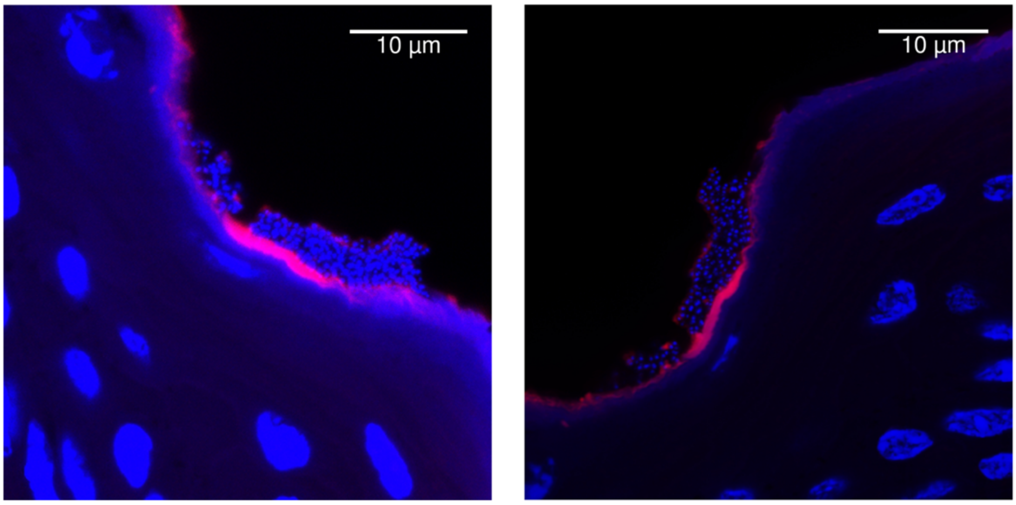

Supplementary Figure 7. Representative confocal images of the cross sections of *S. aureus* USA300 biofilm in the *ex vivo* porcine wound model. The polymeric treatment (Cy5-g-D50) can be observed in red. The nucleic acid of the bacterial cells and mammalian cells was stained with DAPI (blue).

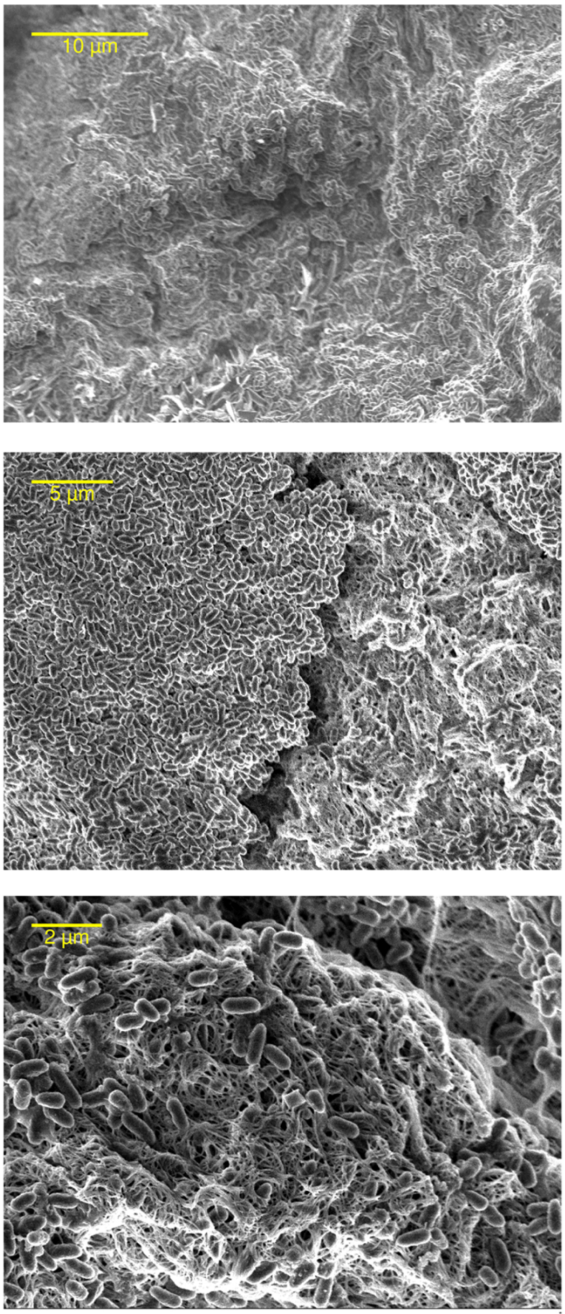

Supplementary Figure 8. Representative SEM micrographs of *P. aeruginosa* PA14 biofilm in the *ex vivo* pig lung model (EVPL).
